# Hydroxycotinine exhibits a stronger association with chronic kidney disease in smokers when compared to cotinine: Evidence from NHANES 2013–2018

**DOI:** 10.18332/tid/201969

**Published:** 2025-03-18

**Authors:** Shili Zhao, Shijing Zheng, Zhiqiang Liu, Yue Xu, Ning Jia, Cihang Lu, Yaning Wang

**Affiliations:** 1 Beijing Hospital of Traditional Chinese Medicine, Capital Medical University, Beijing, People’s Republic of China; 2 Division of Nephrology, Beijing Yanqing Hospital of Traditional Chinese Medicine, Beijing, People’s Republic of China; 3 Division of Endocrinology and Metabolic Diseases, Shengjing Hospital of China Medical University, Shenyang, People’s Republic of China; 4 Department of Nephrology, Binzhou Medical University Hospital, Binzhou, People’s Republic of China

**Keywords:** smoke, cotinine, hydroxycotinine, chronic kidney disease

## Abstract

**INTRODUCTION:**

Smoking is a recognized risk factor for chronic kidney disease (CKD), and cotinine and hydroxycotinine are tobacco metabolites that can be used to quantify smoking. This study evaluated their relationship with CKD in smokers.

**METHODS:**

This secondary dataset analysis is based on National Health and Nutrition Examination Survey (NHANES) data from 2013 to 2018. A cross-sectional examination of a subsample of 2930 adult smokers aged ≥20 years was conducted to investigate the relationship between serum cotinine and its metabolite, hydroxycotinine, and CKD. Linear regression, multivariable-adjusted logistic regression, restrictive cubic splines, and subgroup analysis were utilized.

**RESULTS:**

Serum cotinine and hydroxycotinine levels were significantly elevated in CKD patients compared to the non-CKD population (230.00 vs 212.00 ng/mL, p=0.02 for cotinine; 97.30 vs 74.70 ng/mL, p<0.001 for hydroxycotinine). In multivariable-adjusted logistic regression models, cotinine (≥316 ng/mL) showed a positive association solely with renal insufficiency (adjusted odds ratio, AOR=1.53; 95% CI: 1.07–2.17). In contrast, hydroxycotinine (≥124 ng/mL) was independently associated with three CKD indices: CKD diagnosis (AOR=1.61; 95% CI: 1.06–2.43), renal insufficiency (AOR=2.07; 95% CI: 1.33–3.23), and albuminuria (or proteinuria) (AOR=1.61; 95% CI: 1.06–2.43). Restricted cubic spline analyses revealed nonlinear dose-response relationships: hydroxycotinine exhibited broader negative associations with both eGFR and uACR (p<0.001), while cotinine showed threshold-dependent correlations with CKD risk (positive <180 ng/mL, attenuated above). Subgroup analyses further indicated that hydroxycotinine consistently correlated with CKD across demographics (e.g. males, age <60 years, obesity), whereas cotinine's associations were more limited, with no significant interaction effects observed (p for interaction >0.05).

**CONCLUSIONS:**

Elevated serum concentrations of cotinine and hydroxycotinine are positively associated with low glomerular filtration rate, albuminuria, and CKD in smokers, with hydroxycotinine demonstrating a stronger correlation. Smoking is established as a heightened risk factor for CKD, thus avoidance or reduction of smoking is strongly recommended.

## INTRODUCTION

Smoking constitutes a substantial risk factor for mortality and numerous severe diseases, demanding unwavering attention within the purview of public health^1^. As of 2021, the global population of smokers has surpassed one billion^2^. Meanwhile, the global burden of chronic kidney disease (CKD) continues to escalate. Between 1990 and 2019, the global incidence of CKD cases more than doubled, surging from 7.8 million to 18.99 million^3^. Nevertheless, the overall prevalence of CKD in certain developed nations has exhibited sustained stability in recent years^4-6^.

Smoking constitutes an autonomous risk factor for CKD occurrences^7^. Smoking is believed to exhibit positive correlations with CKD risk, albuminuria, increased GFR, and decreased GFR^8^. Nicotine represents the principal psychoactive compound in both tobacco and e-cigarettes, while cotinine serves as a nicotine metabolite, subsequently undergoing conversion into hydroxycotinine^2,9^. Cotinine can persist in the bloodstream for up to 48 hours, serving as a biomarker for the verification of self-reported smoking habits and exposure to passive smoking^10,11^. Prolonged nicotine exposure may partially contribute to CKD progression and exacerbate renal damage^12,13^. Prior investigations have identified a negative association between serum cotinine levels and renal function, yet research on its relationship with CKD is scarce, and there is a notable absence of pertinent studies on hydroxycotinine^14,15^.

This study attempts to investigate the association between hydroxycotinine and parameters such as renal function, urinary protein, and the risk of CKD. It also aims to compare these findings with those of cotinine.

## METHODS

### Study design and population

This is a secondary dataset analysis of study participants drawn from the National Health and Nutrition Examination Survey (NHANES), encompassing three survey cycles: 2013–2014, 2015–2016, and 2017–2018, accessible at the NHANES website^16^. Participants aged >18 years who reported smoking within the past five days were included, while those with missing data for serum creatinine, urine creatinine, urinary albumin, serum cotinine, hydroxycotinine, and pregnant women were excluded. Ultimately, the analysis incorporated 2930 individuals. Detailed information regarding the inclusion and exclusion process is provided in Supplementary file Figure 1. All data for this project are available and comply with NCHS Ethics Review Board Approval.

### The laboratory methodology of serum creatinine, cotinine, hydroxycotinine, urinary creatinine and urinary albumin

Urine and centrifuged serum samples were appropriately preserved at freezing temperatures (-20°C) for subsequent analysis. The quantification of serum cotinine and hydroxycotinine was performed using isotope-dilution high-performance liquid chromatography combined with atmospheric pressure chemical ionization tandem mass spectrometry. Creatinine concentrations in both serum and urine were determined via the Jaffe rate method, and urine albumin concentration was ascertained using fluorescent immunoassay (FIA). Comprehensive laboratory methods and quality assurance protocols are outlined in the laboratory procedures manual for the NHANES study^17^.

### Diagnostic criteria

These diagnostic criteria were based on the KDIGO 2021 Clinical Practice Guidelines.

Renal insufficiency: eGFR <90 mL/min/1.73 m^2^.

eGFR = 141× min(Scr/κ, 1)^α^ × max(Scr/κ, 1)^-1.209^ × 0.993 × Age × [1.018 if female] × [1.159 if Black], κ was 0.7 for women and 0.9 for men, α was -0.329 for women and -0.411 for men, and min indicates the minimum of Scr/κ or 1, and max indicates the maximum of Scr/κ or 1.^18^

Albuminuria: Urinary albumin creatinine ratio (uACR) ≥30 mg/g^19^.

uACR = urinary albumin/urinary creatinine.

CKD was diagnosed using the KDIGO standard, and a diagnosis was established if at least one of the following criteria is met^19^: eGFR <60 mL/min/1.73 m^2^; eGFR ≥60 mL/min/1.73 m^2^ and uACR ≥30 mg/g.

### Covariates

Structured questionnaires, encompassing demographic and social characteristics (e.g. age, sex, education level, marital status, and poverty ratio level), lifestyle factors (e.g. alcohol consumption status categorized as follows: ‘never’ for individuals with less than 12 drinks in their lifetime, ‘mild or moderate drinkers’ for those with 14 drinks or fewer per week for men or 7 drinks or fewer per week for women, and ‘heavy drinkers’ for those exceeding these limits; leisure-time physical activity level; and dietary habits assessed using the Healthy Eating Index score), and medical history, were administered by trained interviewers. Medical personnel conducted physical examinations, encompassing measurements of height, weight, blood pressure, blood glucose, and glycosylated hemoglobin levels.

### Statistical analysis

All analyses were performed with the application of weighted exam sample weights. For the handling of missing data, multiple imputation was performed utilizing the random forest method^20^, covering the following covariates with missing values: body mass index (BMI) (30 records), education (73 records), income (292 records), marital status (73 records), alcohol consumption status (377 records), physical activity (4 records), and Healthy Eating Index score (249 records), and the specific details are presented in Supplementary file Figures 2 and 3. The scatter plot for linear regression illustrates the correlation between cotinine and hydroxycotinine. Cotinine and hydroxycotinine were stratified into quartiles to investigate their associations with CKD. Logistic regression models were employed to estimate the odds ratios (ORs) for disease-related risk analysis. This analysis utilized three binary logistic regression models: crude, Model 1 (adjusted for fundamental demographic factors), and Model 2 (additional adjustment for lifestyle and health-related factors). A histogram is employed to assess the extent to which a continuous variable approximates a normal distribution (Supplementary file Figure 4). Categorical variables are presented as percentages with changes evaluated using a 95% confidence interval, while continuous variables are expressed as either means or medians, and their changes were assessed with a 95% confidence interval or interquartile range, as applicable. Disparities between categorical variables were assessed using chi-squared tests and Fisher’s exact tests, while differences between continuous variables were evaluated using analysis of variance (ANOVA). Dose-effect analysis was conducted through the application of restricted cubic splines (RCS)[n(knots)=5]. Trend analysis was employed to assess the trend relationship between the quartile distribution of research variables and CKD. Furthermore, multiplicative interaction analysis was utilized to investigate the interaction between research variables and covariates concerning the risk of CKD. The data analysis was executed in R (version 4.3.1), primarily employing the survey, *nhanesR*, *rms*, and *mice* packages. Statistical significance was established at a p<0.05.

## RESULTS

### General characteristics of the participants

A total of 2930 subjects were included in this study, with 1571 males and 1359 females, with an average age of 43.52 years (Table 1). The frequency of CKD was 12.34 (10.53–14.14), as shown in Table 1. Serum cotinine levels in CKD patients showed a slight elevation compared to those in the non-CKD population (230.00 ng/mL vs 212.00 ng/mL, p=0.02), while hydroxycotinine levels exhibited a notable increase in CKD patients in contrast to non-CKD patients (97.30 ng/mL vs 74.70 ng/mL, p<0.001). Variances were observed in age, power ratio level, marital status, alcohol consumption, leisure-time physical activity level, self-reported health status, and self-reported chronic diseases between CKD patients and non-CKD patients. Conversely, the distinctions in gender, BMI, ethnicity, education level, and healthy eating index score between the two groups did not reach statistical significance.

**Table 1:** Baseline characteristics of CKD and non-CKD populations

*Characteristics*	*Total* *% (95% CI)*	*Non-CKD* *% (95% CI)*	*CKD* *% (95% CI)*	*p*
**Total,** n (%)	2930 (100)	2470 (87.66)	460 (12.34)	
**Age** (years), mean (SD)	43.52 (42.57–44.48)	42.16 (41.17–43.15)	53.18 (51.16–55.20)	**<0.001**
**BMI** (kg/m^2^), mean (SD)	28.67 (28.25–29.09)	28.58 (28.16–29.01)	29.26 (28.44–30.09)	0.10
**eGFR** (mL/min/1.73 m^2^), mean (SD)	98.75 (97.72–99.79)	100.66 (99.83–101.49)	85.21 (81.39–89.04)	**<0.001**
**Cotinine** (ng/mL), median (IQR)	213.00 (118.00–308.00)	212.00 (110.00–307.00)	230.00 (152.00–311.00)	**0.02**
**Hydroxycotinine** (ng/mL), median (IQR)	78.50 (34.70–127.00)	74.70 (32.70–123.00)	97.30 (51.20–158.00)	**<0.001**
**Urinary albumin creatinine ratio (**mg/g), median (IQR)	7.09 (4.72–13.46)	6.49 (4.48–10.26)	54.50 (32.81–125.64)	**<0.001**
**Women**	46.37 (41.82–50.92)	45.78 (43.81–47.75)	50.54 (44.48–56.59)	0.14
**Ethnicity**				**0.04**
Non-Hispanic White	65.66 (58.16–73.15)	66.15 (62.41–69.88)	62.18 (55.06–69.30)	
Non-Hispanic Black	13.71 (11.56–15.87)	13.08 (10.74–15.42)	18.20 (13.80–22.61)	
Mexican American	7.05 (5.15–8.95)	7.20 (5.28–9.11)	6.03 (3.28–8.79)	
Other	13.58 (11.76–15.40)	13.58 (11.59–15.56)	13.59 (10.24–16.93)	
**Education level**				0.12
Lower than high school	19.97 (17.08–22.86)	19.33 (16.93–21.73)	24.52 (18.83–30.20)	
High school or equivalent	32.65 (28.74–36.55)	32.54 (29.67–35.41)	33.40 (27.81–38.99)	
College or higher	47.38 (43.50–51.26)	48.13 (45.05–51.21)	42.08 (35.54–48.63)	
**Poverty ratio level**				**<0.001**
0–1.0	26.36 (22.46–30.26)	25.07 (22.02–28.13)	35.49 (31.23–39.74)	
1.1–3.0	44.51 (39.85–49.16)	44.35 (40.92–47.78)	45.62 (40.14–51.10)	
>3.0	29.14 (25.40–32.87)	30.58 (26.66–34.49)	18.89 (13.32–24.47)	
**Marital status**				**<0.001**
Married	52.38 (47.36–57.41)	52.82 (50.00–55.63)	49.32 (43.83–54.81)	
Separated	22.43 (19.58–25.29)	21.21 (18.91–23.51)	31.12 (26.33–35.91)	
Never married	25.18 (22.59–27.78)	25.97 (23.48–28.47)	19.56 (14.69–24.44)	
**Alcohol drinking**				**<0.001**
Non-drinker	11.64 (10.15–13.13)	10.45 (9.09–11.81)	20.08 (16.01–24.16)	
Low to moderate drinker	44.57 (40.98–48.16)	44.62 (42.18–47.05)	44.21 (38.78–49.64)	
Heavy drinker	43.79 (39.11–48.48)	44.93 (42.32–47.55)	35.71 (30.99–40.42)	
**Leisure time physical activity level** (times/week)				**<0.001**
0	56.57 (51.67–61.48)	55.02 (52.51–57.53)	67.60 (61.51–73.68)	
1–2	13.92 (11.72–16.13)	14.80 (12.60–16.99)	7.73 (4.51–10.94)	
≥3	29.50 (26.18–32.82)	30.18 (27.57–32.80)	24.68 (19.35–30.00)	
**Healthy Eating Index score**				0.38
Quarter 1	39.69 (35.54–43.85)	39.98 (37.20–42.76)	37.66 (32.53–42.80)	
Quarter 2	28.11 (25.42–30.79)	27.87 (26.28–29.47)	29.76 (24.15–35.37)	
Quarter 3	21.50 (19.14–23.86)	21.13 (19.22–23.05)	24.09 (19.53–28.64)	
Quarter 4	10.71 (9.13–12.28)	11.02 (9.26–12.78)	8.49 (5.17–11.82)	
**Self-reported health**				**<0.001**
Very good to excellent	28.92 (25.84–32.00)	26.88 (24.69–29.06)	43.42 (35.83–51.02)	
Good	41.44 (37.32–45.57)	42.13 (39.46–44.80)	36.54 (30.19–42.89)	
Poor to fair	29.64 (26.80–32.47)	30.99 (28.51–33.47)	20.04 (13.18–26.89)	
**Self-reported chronic diseases**				
Diabetes	12.15 (10.26–14.05)	9.69 (8.20–11.18)	29.68 (25.21–34.15)	**<0.001**
Hypertension	38.80 (35.01–42.60)	34.72 (31.89–37.56)	67.79 (62.32–73.27)	

IQR: interquartile range.

### Linear relationship between cotinine and hydroxycotinine

**Figure 1:**
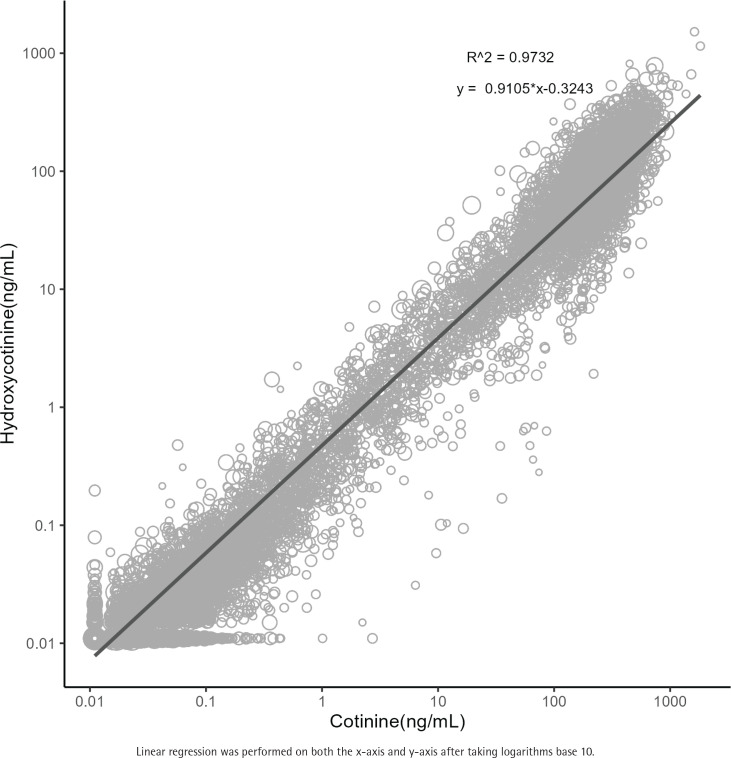
Scatter plots and trend lines of cotinine and hydroxycotinine

Figure 1 illustrates a strong linear relationship between cotinine and hydroxycotinine among smokers (β=0.9105, R^2^=0.9732).

### The associations between serum levels of cotinine and hydroxycotidine and renal dysfunction

Serum cotinine and hydroxycotinine levels were categorized into quartiles (Q1, Q2, Q3, Q4) for analysis. Table 2 presents an investigation into the relationship between cotinine and hydroxycotinine levels and three renal conditions: CKD, albuminuria, and renal insufficiency. This analysis utilized three binary logistic regression models: crude, Model 1 (adjusted for fundamental demographic factors), and Model 2 (additional adjustment for lifestyle and health-related factors).

**Table 2:** Univariate and multivariate logistic regression analysis of cotinine and hydroxycotinine

	*Quartile*	*Crude model*		*Model 1*		*Model 2*	
		*OR (95% CI)*	*p*	*AOR (95% CI)*	*p*	*AOR (95% CI)*	*p*
**Chronic kidney disease**							
**Cotinine**	Q1 ®	1		1		1	
	Q2	1.96 (1.37–2.82)	**<0.001**	1.59 (1.06–2.40)	**0.03**	1.52 (0.99–2.32)	**<0.05**
	Q3	1.65 (1.18–2.31)	**0.004**	1.35 (0.91–2.01)	0.13	1.25 (0.80–1.95)	0.31
	Q4	1.64 (1.07–2.49)	**0.02**	1.26 (0.79–2.00)	0.32	1.19 (0.73–1.94)	0.47
	p for trend		0.09		0.67		0.91
**Hydroxycotinine**	Q1 ®	1		1		1	
	Q2	1.06 (0.70–1.59)	0.78	0.96 (0.61–1.51)	0.85	0.88 (0.55–1.42)	0.59
	Q3	1.28 (0.84–1.94)	0.24	1.09 (0.69–1.73)	0.71	0.96 (0.62–1.61)	0.85
	Q4	2.27 (1.59–3.24)	**<0.001**	2.02 (1.32–3.07)	**0.002**	1.61 (1.06–2.43)	**0.03**
	p for trend		**<0.001**		**0.002**		**0.02**
**Albuminuria**							
**Cotinine**	Q1 ®	1		1		1	
	Q2	1.82 (1.26–2.64)	**0.002**	1.53 (1.02–2.27)	**0.04**	1.46 (0.96–2.22)	0.07
	Q3	1.72 (1.20–2.46)	**0.004**	1.48 (1.00–2.20)	**0.05**	1.38 (0.88–2.17)	0.15
	Q4	1.66 (1.07–2.59)	**0.03**	1.39 (0.85–2.25)	0.18	1.32 (0.79–2.21)	0.28
	p for trend		**<0.05**		0.29		0.44
**Hydroxycotinine**	Q1 ®	1		1		1	
	Q2	1.06 (0.70–1.59)	0.78	0.96 (0.61–1.51)	0.85	0.88 (0.55–1.42)	0.59
	Q3	1.28 (0.84–1.94)	0.24	1.09 (0.69–1.73)	0.71	0.94 (0.62–1.64)	0.85
	Q4	2.28 (1.60–3.24)	**<0.001**	2.02 (1.32–3.07)	**0.002**	1.61 (1.06–2.43)	**0.03**
	p for trend		**<0.001**		**0.002**		**0.02**
**Renal insufficiency**							
**Cotinine**	Q1 ®	1		1		1	
	Q2	1.42 (1.07–1.89)	**0.02**	1.08 (0.77–1.51)	0.65	1.11 (0.78–1.59)	0.53
	Q3	1.84 (1.36–2.50)	**<0.001**	1.42 (0.96–2.11)	0.08	1.47 (0.97–2.21)	0.07
	Q4	1.88 (1.39–2.54)	**<0.001**	1.47 (1.03–2.10)	**0.04**	1.53 (1.07–2.17)	**0.02**
	p for trend		**<0.001**		**0.02**		**0.01**
**Hydroxycotinine**	Q1 ®	1		1		1	
	Q2	1.24 (0.83–1.86)	0.29	1.04 (0.66–1.65)	0.85	1.10 (0.69–1.77)	0.66
	Q3	2.27 (1.54–3.35)	**<0.001**	1.61 (1.06–2.45)	**0.03**	1.66 (1.08–2.56)	**0.02**
	Q4	2.62 (1.79–3.85)	**<0.001**	2.00 (1.30–3.08)	**0.003**	2.07 (1.33–3.23)	**0.003**
	p for trend		**<0.001**		**<0.001**		**<0.001**

Cotinine: Q1, <114 ng/mL; Q2, 114–211 ng/mL; Q3, 212–315 ng/mL; Q4, ≥316 ng/mL. Hydroxycotinine: Q1, <31 ng/mL; Q2, 31–72 ng/mL; Q3, 73–123 ng/mL; Q4, ≥124 ng/mL. Crude model: univariate logistic regression model. AOR: adjusted odds ratio. Model 1: adjusted for baseline age, sex, body mass index, race, education level, marital status, family income–poverty ratio level, and drinking status. Model 2 additionally adjusted for leisure-time physical activity level, healthy eating index scores, self-reported health status and baseline history of diabetes and hypertension. ® Reference categories.

In the unadjusted model, cotinine exhibited a significant association with CKD, showing an odds ratio (OR) of 1.64 (95% CI: 1.07–2.49, p=0.02) for Q4 compared to Q1. However, this association lost significance in Model 1 (Q4, AOR=1.26; 95% CI: 0.79–2.00, p=0.32) and Model 2 (Q4, AOR=1.19; 95% CI: 0.73–1.94, p=0.47). Conversely, hydroxycotinine exhibited a more robust association with CKD, especially in Model 2 (AOR=1.61; 95% CI: 1.06–2.43, p=0.03).

In the unadjusted model, cotinine demonstrated a notable association with albuminuria (OR=1.82; 95% CI: 1.26–2.64, p=0.002). Hydroxycotinine presented a more pronounced association with albuminuria, especially in Model 2, displaying a trend with a p=0.02. Cotinine displayed a significant association with renal insufficiency in the unadjusted model, revealing an OR of 1.42 (95% CI: 1.07–1.89, p=0.02). However, this association progressively declined in Model 1 and Model 2, with a notable reduction in Model 2, resulting in a trend with a p=0.01. In contrast, hydroxycotinine consistently demonstrated a more robust association with renal insufficiency across all models, especially in Model 2, showing a trend with a p<0.001.

### Dose-effect relationship

**Figure 2:**
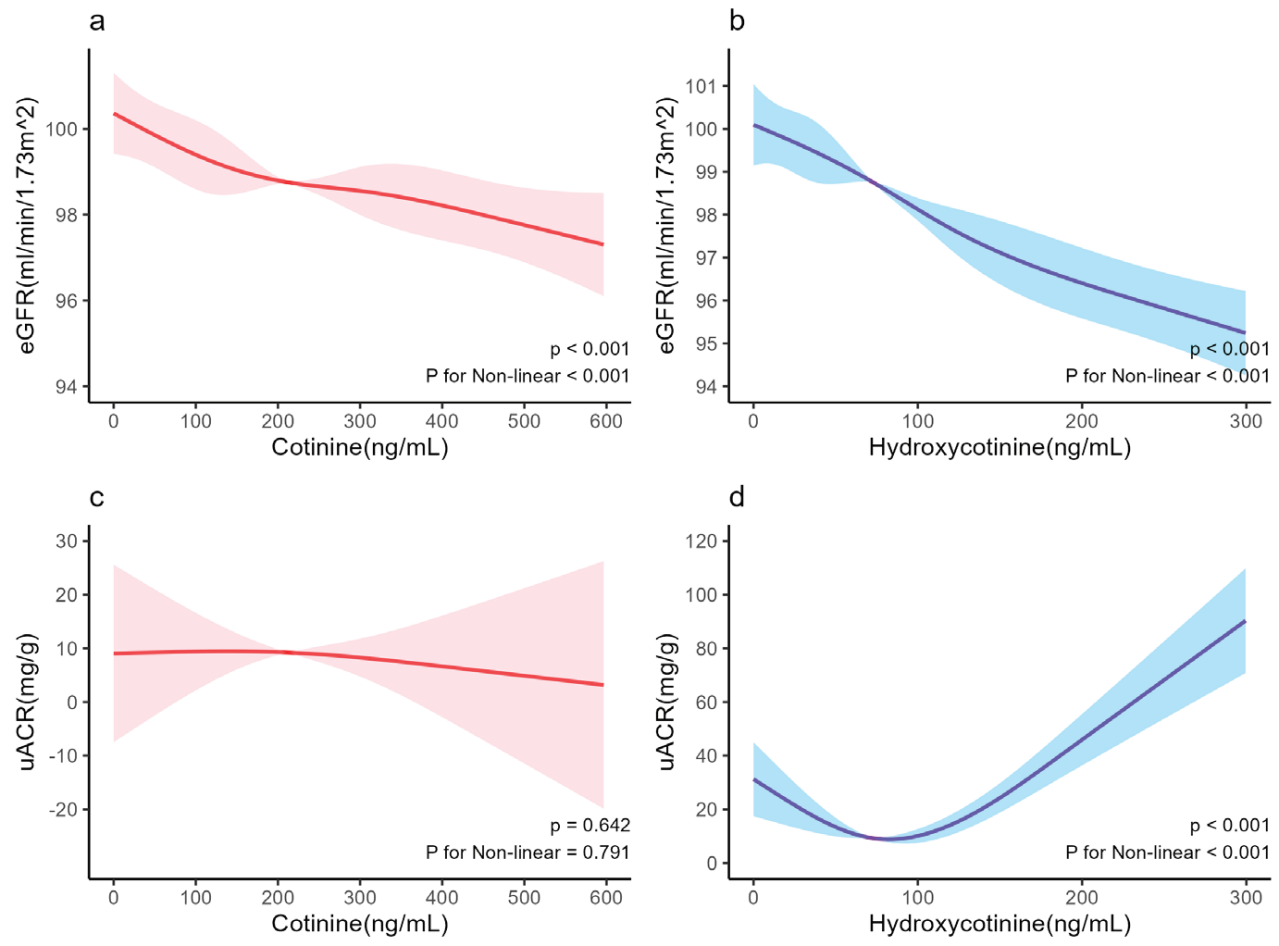
Dose-response associations between eGFR, uACR and serum cotinine, hydroxycotinine level. The red solid line represents the nonlinear relationship between cotinine and the dependent variable, the blue solid line represents the nonlinear relationship between hydroxycotinine and the dependent variable, and the shadow represents the confidence interval

Utilizing RCS fitting linear regression, as depicted in Figure 2, it was evident that both cotinine and hydroxycotinine exhibited a negative correlation with eGFR (p<0.001, p for nonlinearity <0.001). However, notably, only hydroxycotinine displayed a negative correlation with uACR (p<0.001, p for nonlinearity <0.001). Subgroup analysis showed that in males, aged <60 years, BMI ≥30 kg/m^2^, and other populations, the logarithmically transformed base 10 cotinine was negatively correlated with eGFR, while the logarithmically transformed base 10 hydroxycotinine was negatively correlated with eGFR in most subgroups. However, in all subgroups, cotinine and hydroxycotinine were not correlated with uACR (Supplementary file Figures 5–8).

**Figure 3:**
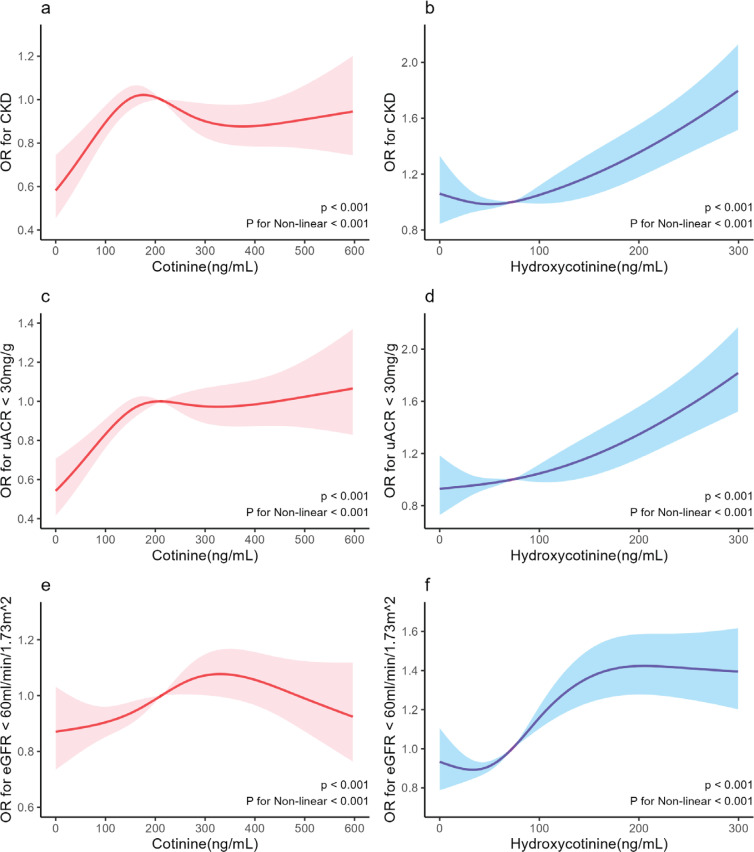
Dose-response associations between serum cotinine and hydroxycotinine level and abnormal renal function (including CKD, albuminuria, renal insufficiency). The red solid line represents the nonlinear relationship between cotinine and the dependent variable, the blue solid line represents the nonlinear relationship between hydroxycotinine and the dependent variable, and the shadow represents the confidence interval

Additionally, RCS fitting logistic regression, presented in Figure 3, indicated intriguing findings. Specifically, when serum cotinine levels were <180 ng/mL, a positive correlation emerged between cotinine concentration and the risk of CKD and albuminuria. However, when cotinine levels were >180 ng/mL, this correlation dissipated, and the relationship between cotinine concentration and the risk of renal insufficiency closely mirrored the trend, except for a greater turning point at 330 ng/mL. Furthermore, it was observed that an increased concentration of serum hydroxycotinine was associated with a significantly heightened risk of CKD, albuminuria, and renal dysfunction in comparison to cotinine (p<0.001, p for nonlinearity<0.001).

### Subgroups analysis

Subgroup analysis, as presented in Table 3, unveiled that an elevation in serum cotinine concentration correlated with an increased risk of CKD among several demographic categories, including males, individuals aged <60 years, those with obesity, non-Hispanic Whites, individuals of other ethnic backgrounds, those with a college education or higher, individuals with poverty ratio levels >1.0, heavy drinkers, those with high scores on the health eating index, respondents reporting good health or better, and individuals with hypertension (OR>1, p<0.05). It is worth noting that in comparison to cotinine, the concentration of hydroxycotinine exhibited a positive correlation with the risk of CKD within a more extensive subgroup of the population, with a notably stronger trend (p<0.05). Importantly, concerning the risk of CKD, neither cotinine nor its metabolite hydroxycotinine demonstrated significant interactions with the grouping variables (p>0.05). Subgroup analysis shows that the elevation of cotinine and hydroxylated cotinine in males was associated with an increased risk of CKD, albuminuria, and renal dysfunction. Elevated levels of cotinine in individuals aged <60 years were associated with an increased risk of CKD, while elevated levels of hydroxylated cotinine were associated with an increased risk of CKD and albuminuria. Elevated levels of cotinine in individuals with BMI <25 kg/m^2^ were associated with increased risk of CKD and albuminuria, while elevated levels of hydroxycotinine were associated with increased risk of albuminuria (proteinuria) (Supplementary file Figures 9–14).

**Table 3:** Subgroup analysis of serum cotinine and hydroxycotinine levels and CKD risk

*Variables*	*Q2* *AOR (95% CI)*	*p*	*Q3* *AOR (95% CI)*	*p*	*Q4* *AOR (95% CI)*	*p*	*p for trend*	*p for* *interaction*
**Cotinine (Q1: ref.)**								
**Sex**								0.8
Men	1.21 (0.72–2.05)	0.46	1.56 (0.86–2.82)	0.14	1.84 (1.07–3.16)	**0.03**	**0.03**	
Women	1.11 (0.70–1.77)	0.65	1.59 (1.00–2.52)	**<0.05**	1.45 (0.85–2.49)	0.16	0.06	
**Age** (years)								0.07
<60	1.09 (0.74–1.60)	0.64	1.82 (1.18–2.81)	**0.01**	1.82 (1.26–2.63)	**0.003**	**<0.001**	
≥60	1.04 (0.45–2.40)	0.93	0.75 (0.36–1.58)	0.44	0.86 (0.36–2.03)	0.72	0.54	
**BMI** (kg/m^2^)								0.57
<25.0	1.16 (0.57–2.37)	0.67	1.55 (0.73–3.28)	0.24	1.39 (0.63–3.09)	0.4	0.31	
25.0–29.9	1.36 (0.88–2.11)	0.15	1.17 (0.65–2.13)	0.58	1.58 (0.88–2.82)	0.12	0.18	
≥30	1.02 (0.63–1.66)	0.93	1.98 (1.25–3.13)	**0.01**	1.81 (1.00–3.28)	**<0.05**	**0.002**	
**Ethnicity**								0.87
Non-Hispanic White	1.04 (0.64–1.69)	0.86	1.53 (0.95–2.47)	0.08	1.53 (0.99–2.35)	**<0.05**	**0.03**	
Non-Hispanic Black	0.96 (0.51–1.80)	0.88	1.07 (0.55–2.07)	0.82	1.03 (0.55–1.91)	0.93	0.79	
Mexican American	1.27 (0.46–3.53)	0.71	2.28 (0.82–6.33)	0.34	1.48 (0.25–8.88)	0.73	0.24	
Other	1.56 (0.73–3.33)	0.24	2.06 (0.90–4.74)	0.08	3.25 (1.44–7.35)	**0.01**	**0.01**	
**Education level**								0.84
Lower than high school	0.83 (0.39–1.77)	0.61	1.17 (0.46–3.01)	0.73	1.22 (0.45–3.32)	0.69	0.45	
High school or equivalent	1.54 (0.79–3.00)	0.19	2.01 (1.14–3.57)	**0.02**	1.66 (0.93–2.95)	0.08	0.08	
College or higher	1.02 (0.59–1.76)	0.94	1.47 (0.83–2.61)	0.17	1.75 (1.08–2.81)	**0.02**	**0.01**	
**Poverty ratio level**								0.11
0–1.0	0.53 (0.26–1.11)	0.09	1.06 (0.50–2.23)	0.88	0.87 (0.49–1.55)	0.63	0.56	
1.1–3.0	1.65 (1.05–2.59)	**0.03**	2.01 (1.26–3.21)	**0.01**	1.63 (1.10–2.42)	**0.02**	**0.02**	
>3.0	1.11 (0.57–2.16)	0.74	1.65 (0.74–3.69)	0.21	3.04 (1.39–6.67)	**0.01**	**0.01**	
**Alcohol drinking**								0.52
Non-drinker	1.09 (0.39–3.04)	0.86	1.49 (0.40–5.53)	0.53	2.42 (0.86–6.78)	0.09	**0.04**	
Low to moderate drinker	1.16 (0.73–1.83)	0.52	1.47 (0.95–2.26)	0.08	1.28 (0.78–2.10)	0.31	0.19	
Heavy drinker	1.04 (0.60–1.79)	0.9	1.79 (1.01–3.17)	**0.05**	1.83 (1.07–3.12)	**0.03**	**0.01**	
**Leisure time physical activity level** (times/week)								0.87
0	1.17 (0.82–1.65)	0.37	1.69 (1.11–2.58)	**0.02**	1.74 (1.09–2.78)	**0.02**	**0.02**	
1–2	1.26 (0.43–3.69)	0.66	2.19 (0.59–8.05)	0.22	1.18 (0.29–4.91)	0.81	0.53	
≥3	1.21 (0.68–2.17)	0.5	1.65 (0.84–3.26)	0.14	1.96 (0.88–4.35)	0.1	0.07	
**Healthy Eating Index score**								0.35
Quarter 1	0.78 (0.38–1.59)	0.48	1.77 (0.96–3.25)	0.06	1.57 (0.90–2.76)	0.11	**0.01**	
Quarter 2	1.42 (0.81–2.46)	0.21	1.54 (0.71–3.31)	0.26	1.66 (0.89–3.10)	0.11	0.13	
Quarter 3	0.78 (0.40–1.50)	0.44	1.07 (0.49–2.33)	0.86	1.25 (0.55–2.85)	0.58	0.46	
Quarter 4	3.44 (1.27–9.36)	**0.02**	1.71 (0.62–4.71)	0.28	2.54 (1.05–6.17)	**0.04**	0.1	
**Self-reported health**								0.74
Very good to excellent	0.92 (0.50–1.69)	0.78	1.57 (0.89–2.80)	0.12	1.42 (0.81–2.49)	0.21	**<0.05**	
Good	1.43 (0.84–2.41)	0.17	1.70 (0.87–3.34)	0.12	1.93 (1.10–3.40)	**0.02**	**0.02**	
Poor to fair	1.07 (0.55–2.08)	0.84	1.54 (0.84–2.84)	0.16	1.43 (0.79–2.60)	0.23	0.1	
**Hydroxycotinine (Q1: ref.)**								
**Sex**								0.32
Men	1.31(0.76–2.27)	0.31	2.28 (1.24–4.18)	**0.01**	2.24 (1.29–3.90)	**0.01**	**0.001**	
Women	1.01 (0.54–1.89)	0.98	1.43 (0.82–2.50)	0.19	2.23 (1.31–3.81)	**0.01**	**<0.001**	
**Age** (years)								0.69
<60	1.18 (0.71–1.95)	0.51	1.94 (1.20–3.12)	**0.01**	2.32 (1.47–3.67)	**0.001**	**<0.001**	
≥60	0.69 (0.27–1.78)	0.43	1.12 (0.57–2.20)	0.74	1.51 (0.72–3.17)	0.26	**0.05**	
**BMI** (kg/m^2^)								0.76
<25.0	1.06 (0.43–2.64)	0.89	1.70 (0.75–3.84)	0.19	1.65 (0.72–3.78)	0.22	0.13	
25.0–29.9	1.34 (0.72–2.52)	0.34	1.94 (0.95–3.96)	0.07	2.13 (1.20–3.78)	**0.01**	**0.01**	
≥30	1.08 (0.65–1.79)	0.76	1.80 (1.10–2.94)	**0.02**	3.07 (1.78–5.28)	**<0.001**	**<0.001**	
**Ethnicity**								0.53
Non-Hispanic White	1.00 (0.52–1.94)	1	1.53 (0.86–2.74)	0.14	1.92 (1.12–3.27)	**0.02**	**0.003**	
Non-Hispanic Black	0.64 (0.32–1.28)	0.18	1.63 (0.84–3.14)	0.13	1.61 (0.85–3.04)	0.13	**0.01**	
Mexican American	2.79 (1.42–5.48)	0.2	3.18 (1.10–9.20)	0.27	8.16 (2.43–27.41)	0.17	**0.03**	
Other	2.03 (1.06–3.91)	**0.03**	2.91 (1.40–6.05)	**0.01**	3.87 (1.39–10.73)	**0.01**	**0.004**	
**Education level**								0.45
Lower than high school	1.60 (0.87–2.95)	0.12	2.23 (1.10–4.53)	**0.03**	2.95 (1.48–5.86)	**0.004**	**0.004**	
High school or equivalent	1.20 (0.61–2.37)	0.57	2.17 (1.22–3.88)	**0.01**	1.72 (0.99–2.99)	**0.05**	**0.03**	
College or higher	0.97 (0.51–1.84)	0.92	1.46 (0.79–2.70)	0.22	2.38 (1.23–4.58)	**0.01**	**0.01**	
**Poverty ratio level**								0.37
0–1.0	1.18 (0.59–2.36)	0.63	1.47 (0.83–2.61)	0.18	2.76 (1.64–4.64)	**<0.001**	**<0.001**	
1.1–3.0	1.33 (0.76–2.31)	0.3	1.94 (1.19–3.16)	**0.01**	2.43 (1.44–4.10)	**0.002**	**<0.001**	
>3.0	0.83 (0.32–2.11)	0.68	2.00 (0.87–4.62)	0.1	1.64 (0.64–4.18)	0.29	0.1	
**Alcohol drinking**								0.28
Non-drinker	1.87 (0.86–4.07)	0.11	1.99 (0.69–5.73)	0.19	2.55 (1.05–6.21)	**0.04**	0.06	
Low to moderate drinker	0.99 (0.55–1.78)	0.97	2.23 (1.35–3.67)	**0.003**	2.12 (1.31–3.41)	**0.004**	**<0.001**	
Heavy drinker	1.06 (0.59–1.90)	0.85	1.35 (0.73–2.48)	0.32	2.16 (1.20–3.88)	**0.01**	**0.01**	
**Leisure time physical activity level** (times/week)								0.78
0	1.04 (0.66–1.65)	0.85	1.98 (1.27–3.09)	**0.004**	2.15 (1.32–3.52)	**0.004**	**<0.001**	
1–2	1.17 (0.33–4.21)	0.8	1.53 (0.45–5.16)	0.48	1.84 (0.58–5.81)	0.28	0.24	
≥3	1.56 (0.69–3.50)	0.27	1.90 (0.95–3.79)	0.07	2.86 (1.28–6.41)	**0.01**	**0.01**	
**Healthy Eating Index score**								0.95
Quarter 1	0.95 (0.46–1.99)	0.89	2.00 (1.18–3.37)	**0.01**	2.03 (1.09–3.79)	**0.03**	**0.004**	
Quarter 2	1.47 (0.85–2.56)	0.16	1.74 (0.90–3.34)	0.09	2.63 (1.28–5.42)	**0.01**	**0.01**	
Quarter 3	0.95 (0.37–2.46)	0.91	1.26 (0.60–2.61)	0.53	2.19 (0.91–5.27)	0.08	0.07	
Quarter 4	1.35 (0.45–4.04)	0.58	2.94 (1.05–8.25)	**0.04**	2.46 (0.91–6.67)	0.07	**0.04**	
**Self-reported health**								0.77
Very good to excellent	1.13 (0.64–2.01)	0.66	1.55 (0.83–2.91)	0.16	2.23 (1.40–3.53)	**0.002**	**0.005**	
Good	1.29 (0.69–2.39)	0.4	2.02 (1.10–3.71)	**0.03**	2.38 (1.14–4.97)	**0.02**	**0.01**	
Poor to fair	0.99 (0.41–2.37)	0.98	2.04 (0.96–4.37)	0.06	1.72 (0.78–3.81)	0.17	0.06	

Cotinine: Q1, <114 ng/mL; Q2, 114–211 ng/mL; Q3, 212–315 ng/mL; Q4, ≥316 ng/mL. Hydroxycotinine: Q1, <31 ng/mL; Q2, 31–72 ng/mL; Q3, 73–123 ng/mL; Q4, ≥124 ng/mL. AOR: adjusted odds ratio; adjusted for baseline age, sex, race, education level, marital status, family income–poverty ratio level, drinking and smoking status, leisure-time physical activity level, healthy eating index scores, self-reported health status, baseline history of diabetes and hypertension.

## DISCUSSION

Our data reveal that hydroxycotinine displays a more robust association with chronic kidney disease (CKD) when compared to cotinine, although both compounds were correlated with renal function. While prior research has established a negative correlation between serum cotinine and estimated glomerular filtration rate (eGFR), this study investigated the correlation between hydroxycotinine and eGFR, as well as their respective associations with CKD^14,15^.

The study exclusively investigated current smokers due to the significantly reduced serum levels of cotinine and hydroxycotinine in non-smokers and former smokers, with even those exposed to tobacco smoke displaying substantially lower levels compared to current smokers^10^. While urine cotinine concentration typically surpasses serum and saliva cotinine concentration by 2–4 times and is generally considered the most reliable indicator for determining smoking status, it is noteworthy that urine cotinine concentration can be influenced by renal function. Therefore, this investigation chose to utilize serum cotinine concentration to assess the relationship between cotinine levels within the body and renal function. A robust linear relationship was observed between cotinine and hydroxycotinine, the latter being a metabolite of cotinine^21^.

Smoking is significantly correlated with both eGFR and uACR, with the association being prominent in younger populations, as indicated by many studies^22-26^. Persistent smokers face a two-fold or greater risk of developing proteinuria, and smoking cessation can mitigate this risk, as supported by 15 prospective cohort studies, including a meta-analysis of 65064 CKD cases, all arriving at the same conclusion^27,28^. Current smokers were at a higher risk of CKD than former smokers, with both groups exhibiting elevated risks compared to individuals who have never smoked^7^. Notably, this study demonstrates a negative correlation between serum levels of cotinine and hydroxycotinine and eGFR in current smokers, while only hydroxycotinine shows a positive correlation with uACR. These findings align with prior studies in the general population, suggesting that the link between smoking and glomerular hyperfiltration is independent of cotinine and its metabolite hydroxycotinine^14,29^. Both serum cotinine and hydroxycotinine exhibit positive correlations with renal dysfunction, microalbuminuria, and CKD risk, confirming their roles as risk factors, and passive smoking is shown to adversely affect kidney morphology and glomerular filtration rate^30^. Furthermore, a novel correlation between hydroxycotinine and CKD risk, compared to cotinine, has been identified, necessitating well-structured cohort studies for validation and further exploration of hydroxycotinine’s potential role in CKD onset and progression.

Smoking has detrimental effects on individuals with CKD. Cohort studies have unveiled a significant correlation between smoking and an increased risk of renal function deterioration in CKD patients, with this association displaying a dose-dependent impact. Ceasing smoking may delay the progression of CKD and ameliorate unfavorable renal outcomes^31^. In a distinct cohort study involving CKD patients, smoking considerably heightened the risk of both vascular and non-vascular disease incidence and mortality^32^.

Studies on the renal consequences of smoking are limited and have primarily focused on nicotine. Prolonged exposure to nicotine exacerbates acute renal ischemic injury. *In vivo* experiments have shown that nicotine intensifies the extent of renal damage in animal models, encompassing conditions such as acute renal injury, diabetes, acute nephritis, and subtotal nephrectomy^13^. The renal effects of nicotine were primarily attributed to an increased production of reactive oxygen species (oxidative stress) and the activation of pathways that promote fibrosis^12,13,33,34^. Furthermore, nicotine may harm the kidneys by upregulating Grem1 expression, activating non-neuronal nicotinic acetylcholine receptors, and inducing Akt phosphorylation^34-36^. Regrettably, there is currently no published research that directly investigates the mechanisms underlying the effects of cotinine and hydroxycotinine on the kidneys, despite their status as nicotine metabolites. It is hoped that this may become a focal point for future research in related fields.

### Limitations

This study has several limitations. First, the cross-sectional design limited the ability to establish causal relationships between serum cotinine, hydroxycotinine levels, and CKD. Second, reliance on self-reported smoking status and lifestyle factors may have introduced reporting bias. Third, although we adjusted for key confounders (e.g. age, comorbidities), residual confounding from unmeasured variables (e.g. environmental toxin exposure) could persist. Finally, the findings were derived from the NHANES dataset, which may limit their generalizability to non-smokers or populations with distinct genetic or environmental backgrounds. Additionally, the study population was restricted to a US cohort; thus, the results might not be extrapolated to other countries with differing healthcare systems, smoking prevalence patterns, or environmental exposures.

## CONCLUSIONS

Elevated levels of serum cotinine and hydroxycotinine are associated with an increased risk of reduced glomerular filtration rate, microalbuminuria, and CKD in smokers, with hydroxycotinine showing a stronger correlation. Further validation through carefully designed cohort studies and mechanistic experiments is anticipated in the future. Nevertheless, we emphasize that an elevated level of smoking, especially in situations with high levels of cotinine and hydroxycotinine, poses a risk for CKD. Therefore, we advocate for smoking avoidance or reduction.

## Supplementary Material



## Data Availability

The data supporting this research are available from the authors on reasonable request.
