# Supplementary material for: Hydroxycotinine exhibits a stronger association with chronic kidney disease in smokers when compared to cotinine: Evidence from NHANES 2013–2018
Source: Tob Induc Dis. 2025 Mar 18;23:10.18332/tid/201969. doi: 10.18332/tid/201969 (PMC11915096; doi:10.18332/tid/201969)
Supplement: Supplementary file 1 [file TID-23-34-s1.pdf]

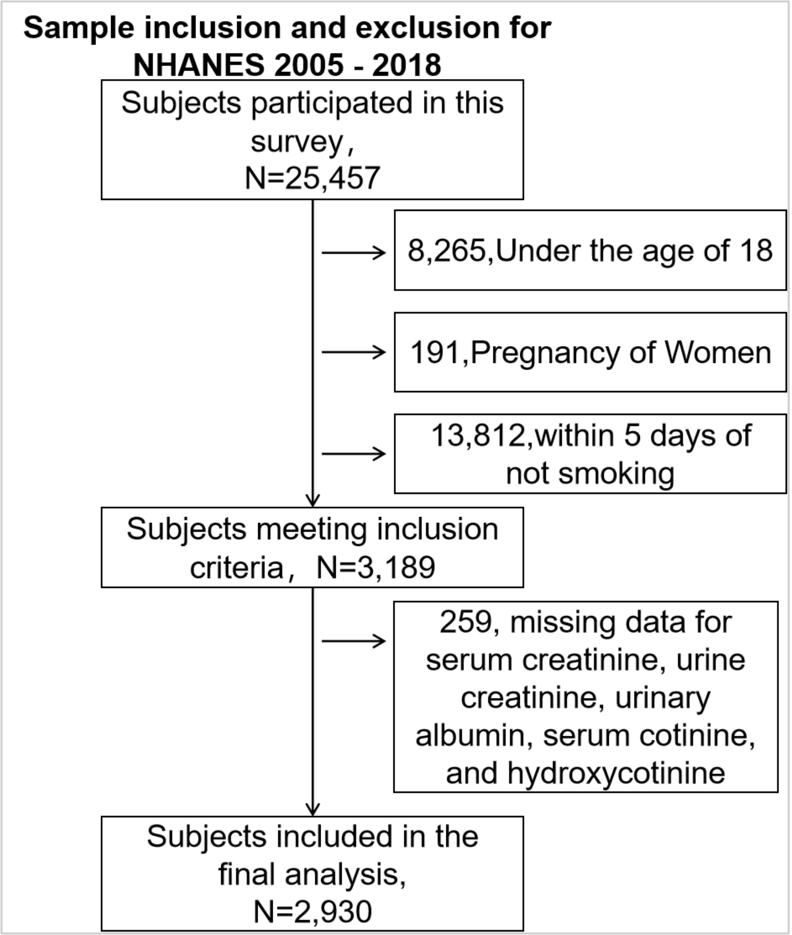

Supplementary Figure 1, The screening process of samples

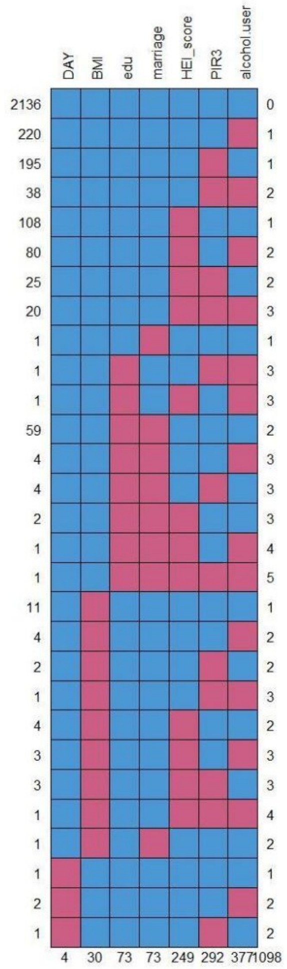

Supplementary Figure 2, Number of missing covariates

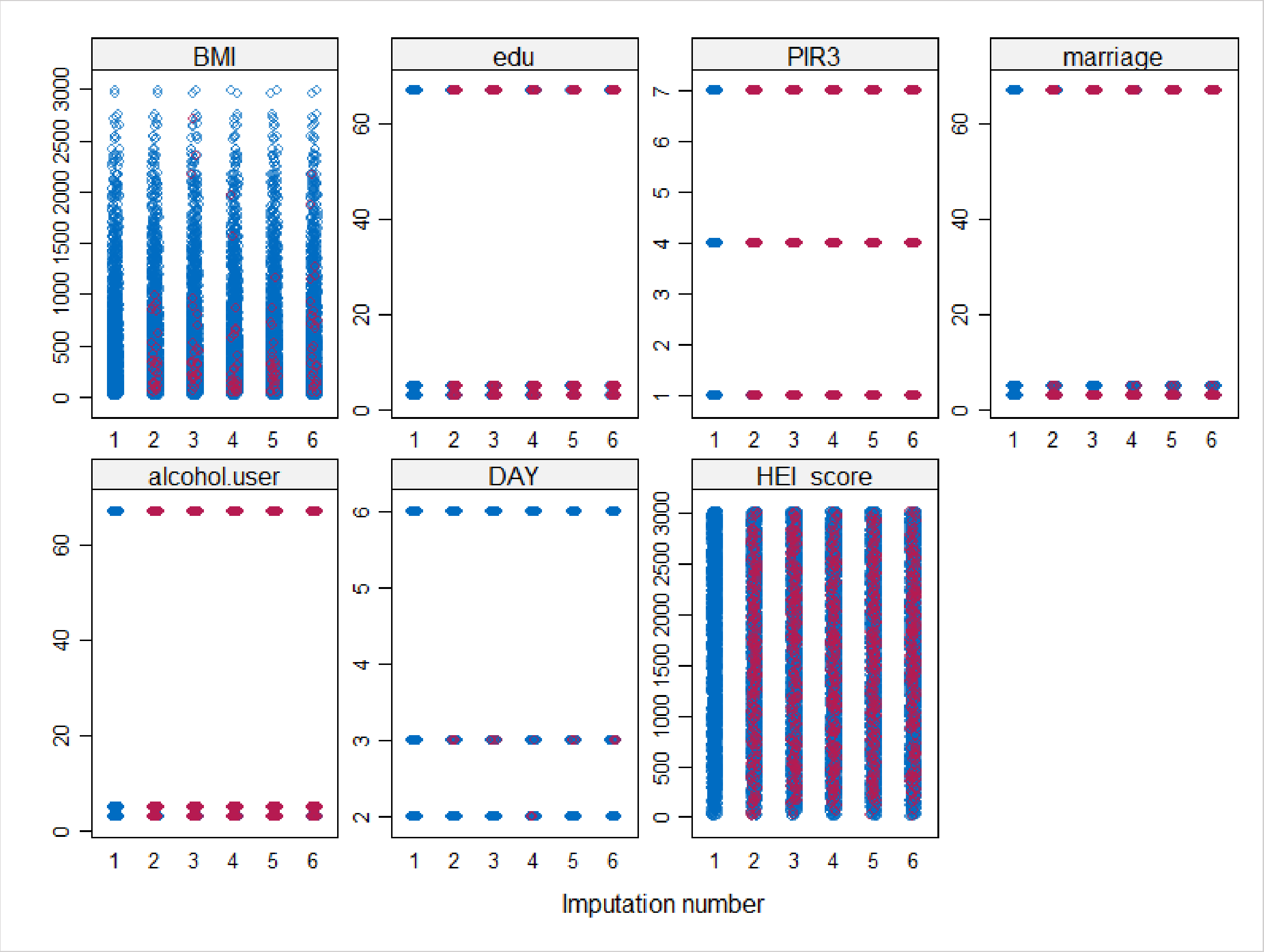

Supplementary Figure 3, Multiple imputation of missing values in covariates

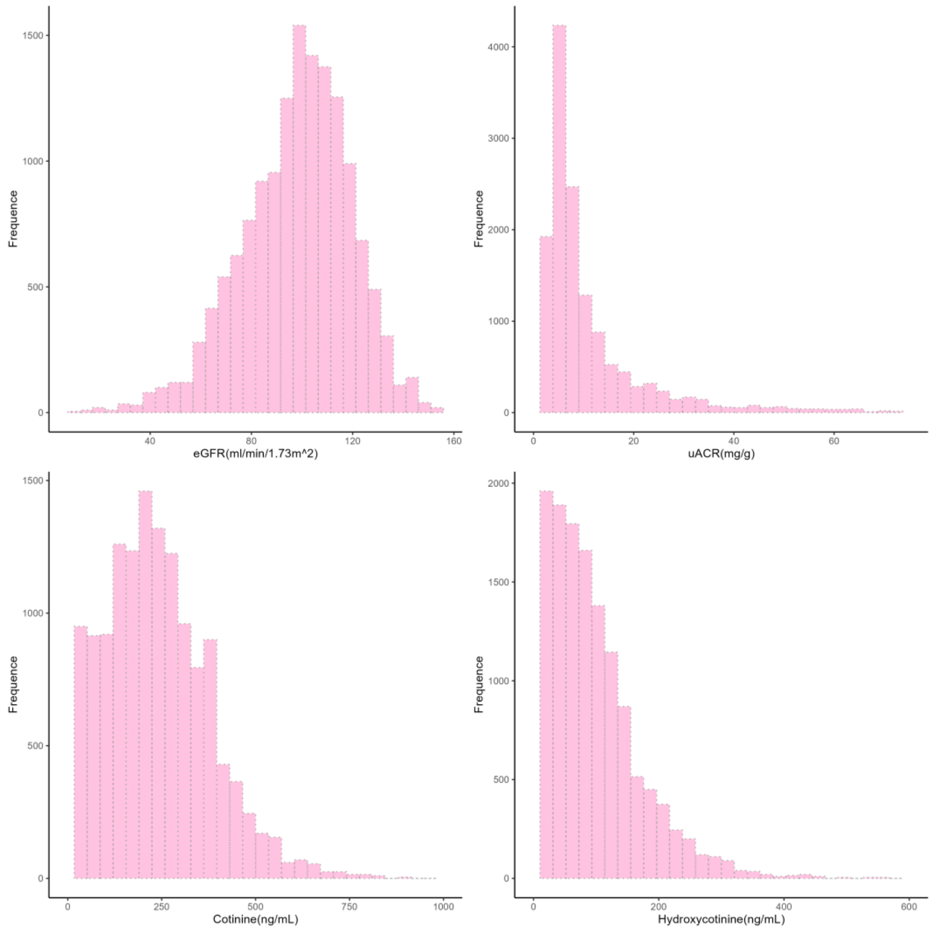

Supplementary Figure 4, Histograms of cotinine, hydroxylated cotinine, eGFR, and uACR

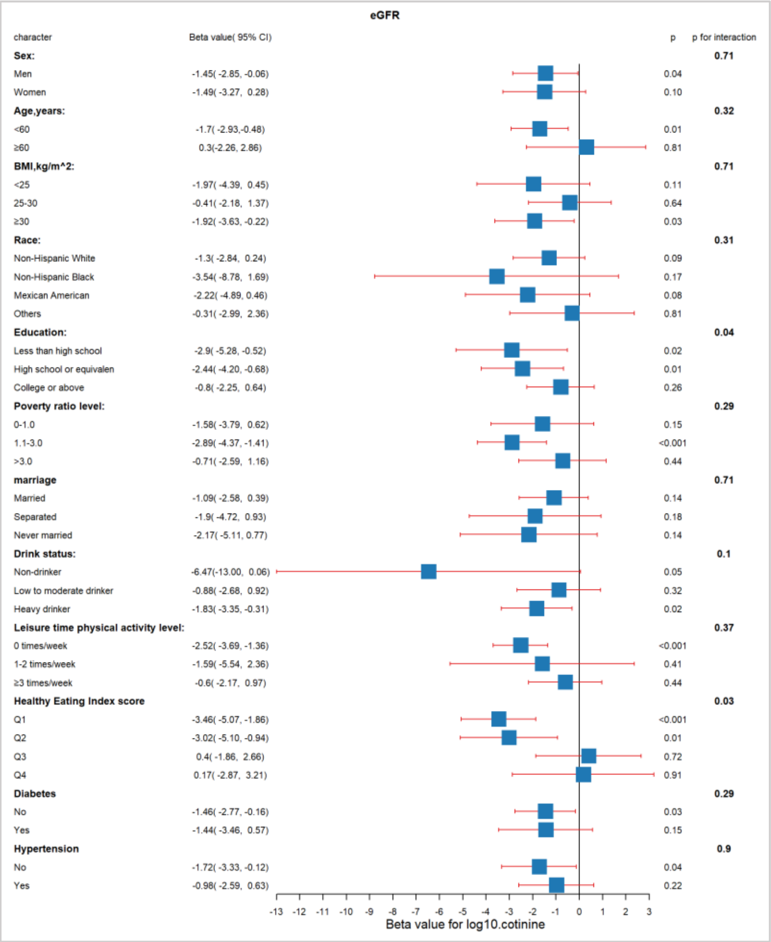

Supplementary Figure 5, Subgroup analysis of generalized linear regression between cotinine and eGFR after logarithmic transformation with a base of 10, adjusted for baseline age, sex, BMI, race, education level, marital status, family income- poverty ratio level, drinking status, leisure-time physical activity level, healthy eating index scores, self-reported health status and baseline history of diabetes and hypertension.

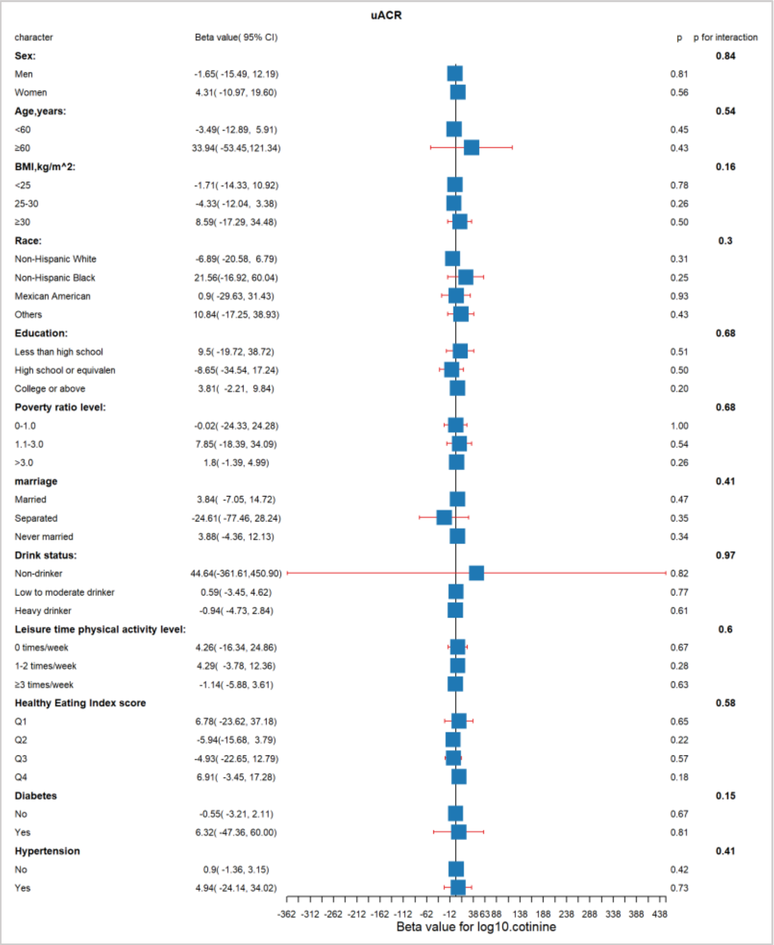

Supplementary Figure 6, Subgroup analysis of generalized linear regression between cotinine and uACR after logarithmic transformation with a base of 10, adjusted for baseline age, sex, BMI, race, education level, marital status, family income- poverty ratio level, drinking status, leisure-time physical activity level, healthy eating index scores, self-reported health status and baseline history of diabetes and hypertension.

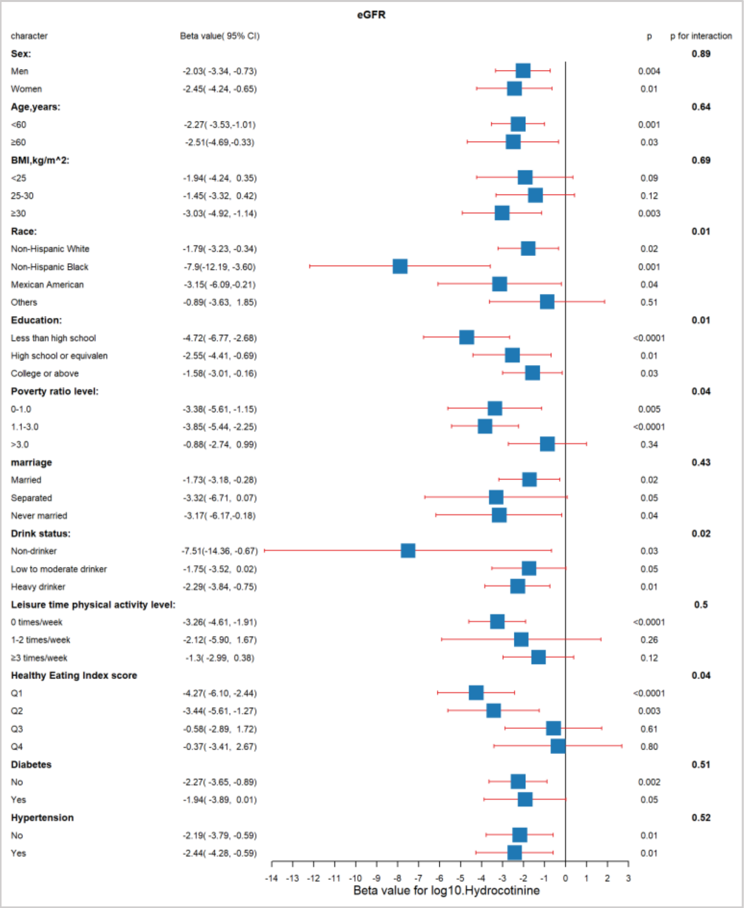

Supplementary Figure 7, Subgroup analysis of generalized linear regression between hydrocotinine and eGFR after logarithmic transformation with a base of 10, adjusted for baseline age, sex, BMI, race, education level, marital status, family income- poverty ratio level, drinking status, leisure-time physical activity level, healthy eating index scores, self-reported health status and baseline history of diabetes and hypertension.

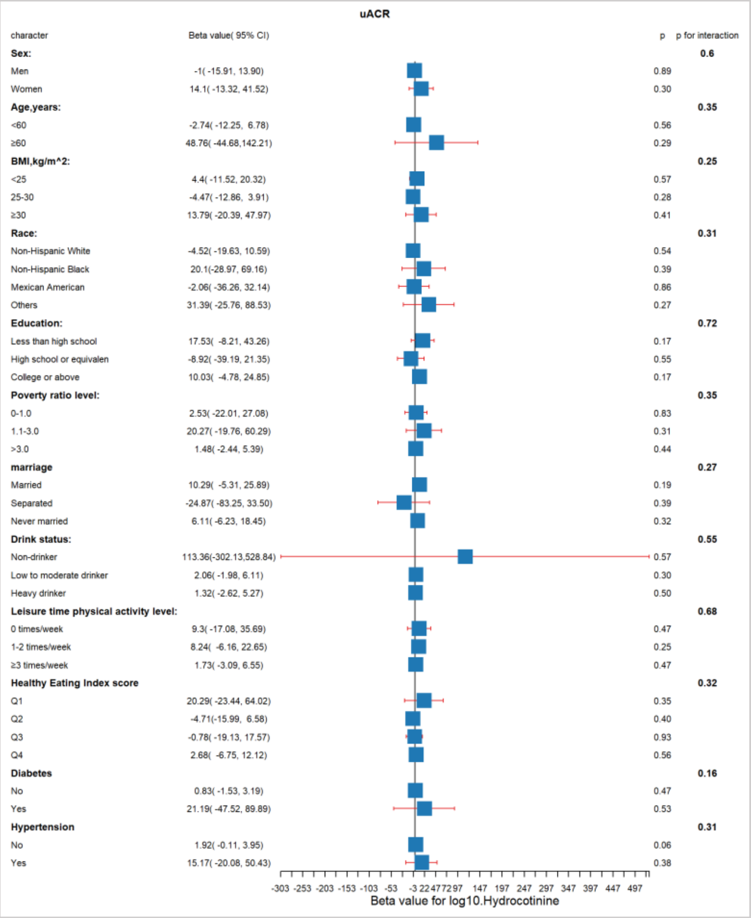

Supplementary Figure 8, Subgroup analysis of generalized linear regression between hydrocotinine and uACR after logarithmic transformation with a base of 10, adjusted for baseline age, sex, BMI, race, education level, marital status, family income- poverty ratio level, drinking status, leisure-time physical activity level, healthy eating index scores, self-reported health status and baseline history of diabetes and hypertension.

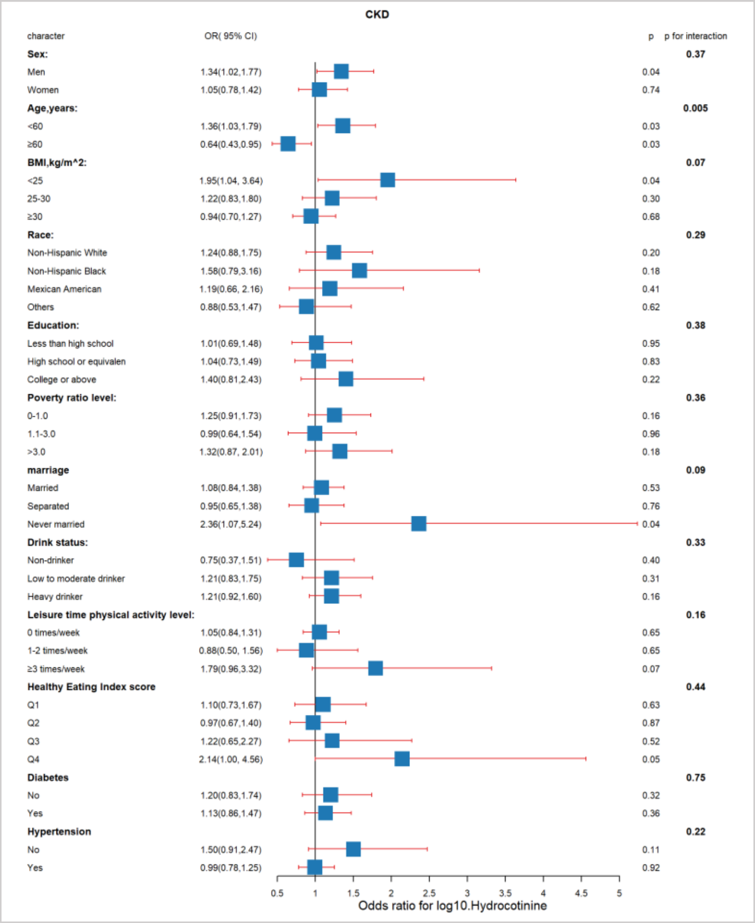

Supplementary Figure 9, Subgroup analysis of logistic regression between cotinine and CKD after logarithmic transformation with a base of 10, adjusted for baseline age, sex, BMI, race, education level, marital status, family income- poverty ratio level, drinking status, leisure-time physical activity level, healthy eating index scores, self-reported health status and baseline history of diabetes and hypertension.

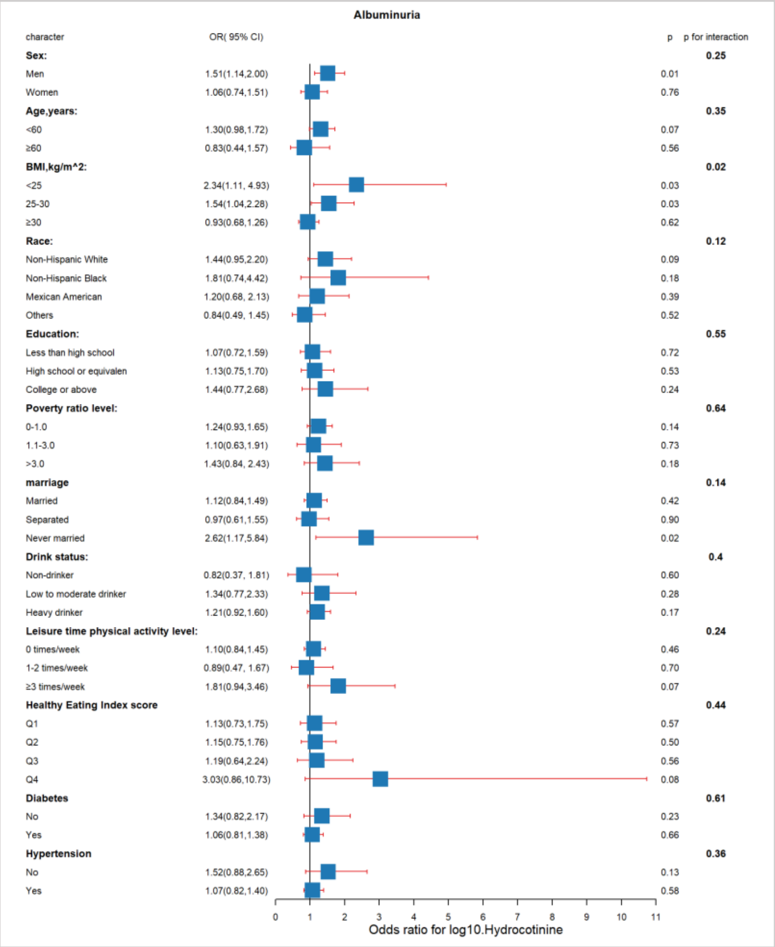

Supplementary Figure 10, Subgroup analysis of logistic regression between cotinine and Albuminuria after logarithmic transformation with a base of 10, adjusted for baseline age, sex, BMI, race, education level, marital status, family income- poverty ratio level, drinking status, leisure-time physical activity level, healthy eating index scores, self-reported health status and baseline history of diabetes and hypertension.

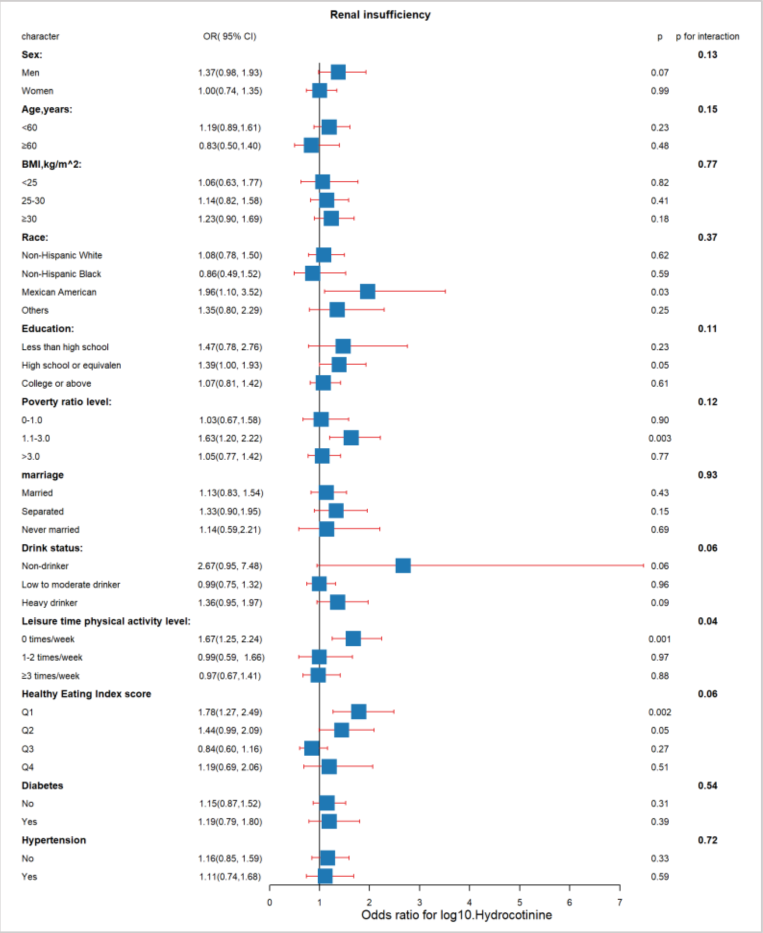

Supplementary Figure 11, Subgroup analysis of logistic regression between cotinine and Renal insufficiency after logarithmic transformation with a base of 10, adjusted for baseline age, sex, BMI, race, education level, marital status, family income- poverty ratio level, drinking status, leisure-time physical activity level, healthy eating index scores, self-reported health status and baseline history of diabetes and hypertension.

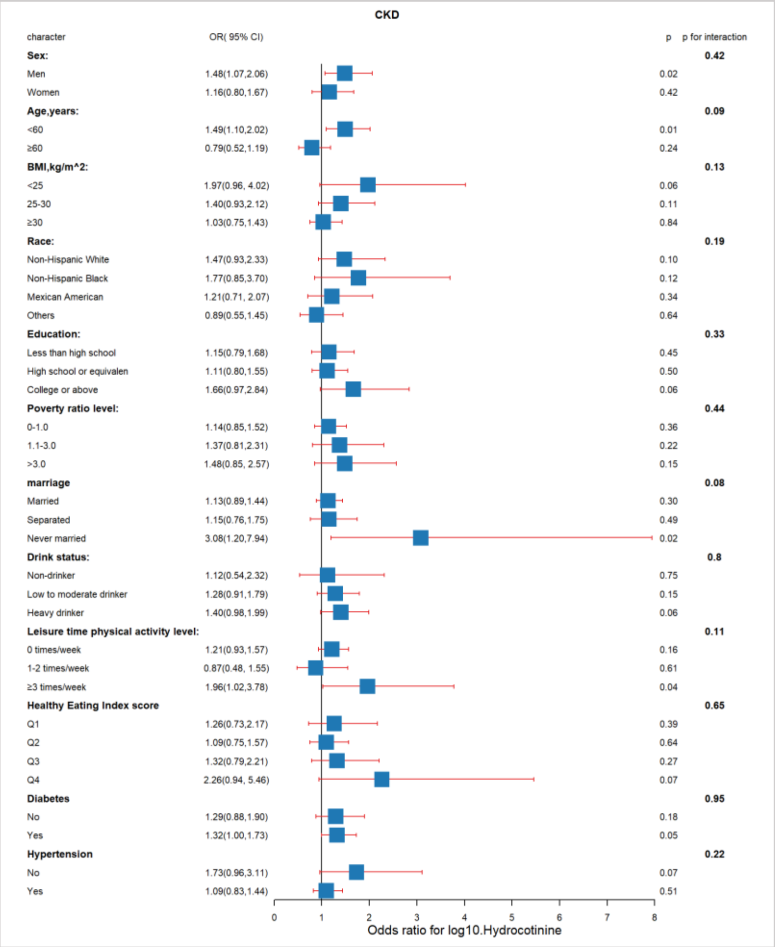

Supplementary Figure 12, Subgroup analysis of logistic regression between hydrocotinine and CKD after logarithmic transformation with a base of 10, adjusted for baseline age, sex, BMI, race, education level, marital status, family income- poverty ratio level, drinking status, leisure-time physical activity level, healthy eating index scores, self-reported health status and baseline history of diabetes and hypertension.

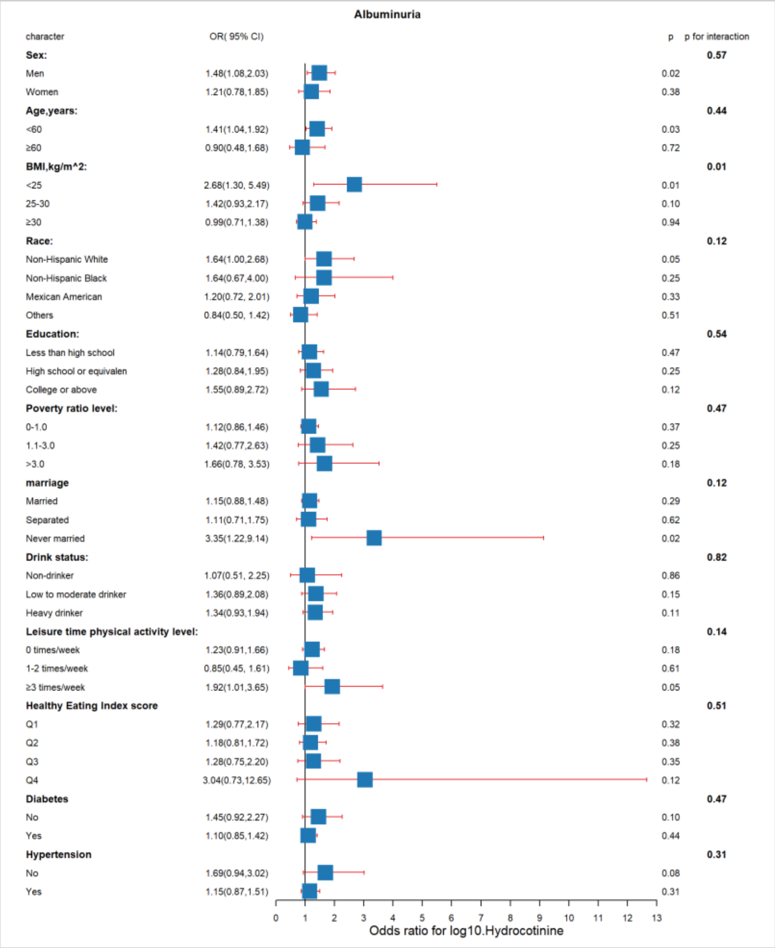

Supplementary Figure 13, Subgroup analysis of logistic regression between hydrocotinine and Albuminuria after logarithmic transformation with a base of 10, adjusted for baseline age, sex, BMI, race, education level, marital status, family income- poverty ratio level, drinking status, leisure-time physical activity level, healthy eating index scores, self-reported health status and baseline history of diabetes and hypertension.

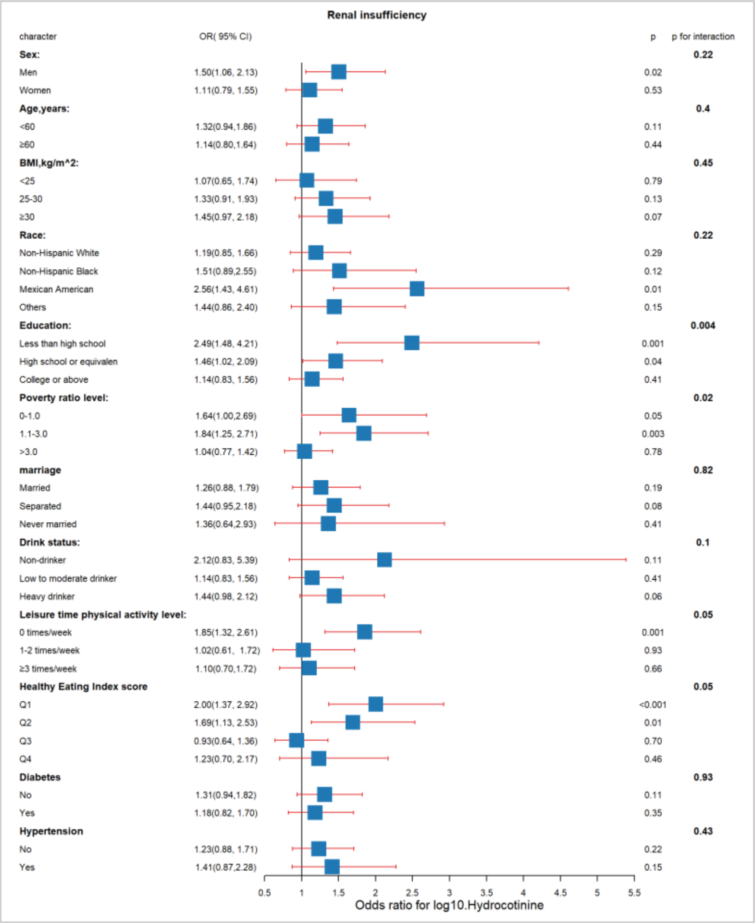

Supplementary Figure 14, Subgroup analysis of logistic regression between hydrocotinine and Renal insufficiency after logarithmic transformation with a base of 10, adjusted for baseline age, sex, BMI, race, education level, marital status, family income- poverty ratio level, drinking status, leisure-time physical activity level, healthy eating index scores, self-reported health status and baseline history of diabetes and hypertension.

	Total (N=2930)	Non-CKD (N=2470,87.66%)	CKD (N=460,12.34%)	*P-value*
Age, years*	43.52(42.57,44.48)	42.16(41.17,43.15)	53.18(51.16,55.20)	**< 0.001**
BMI, kg/m^2*	28.67(28.25,29.09)	28.58(28.16,29.01)	29.26(28.44,30.09)	0.10
eGFR, ml/min/1.73m^2*	98.75(97.72,99.79)	100.66(99.83,101.49)	85.21(81.39, 89.04)	**< 0.001**
Cotinine, ng/mL#	213.00(118.00,308.00)	212.00(110.00,307.00)	230.00(152.00,311.00)	**0.02**
Hydroxycotinine, ng/mL#	78.50(34.70,127.00)	74.70(32.70,123.00)	97.30(51.20,158.00)	**< 0.001**
Urinary albumin creatinine ratio, mg/g#	7.09(4.72,13.46)	6.49(4.48, 10.26)	54.50(32.81,125.64)	**< 0.001**
Women	46.37(41.82,50.92)	45.78(43.81,47.75)	50.54(44.48,56.59)	0.14
Ethnicity:				**0.04**
Non-Hispanic white	65.66(58.16,73.15)	66.15(62.41,69.88)	62.18(55.06,69.30)	
Non-Hispanic black	13.71(11.56,15.87)	13.08(10.74,15.42)	18.20(13.80,22.61)	
Mexican American	7.05(5.15, 8.95)	7.20(5.28,9.11)	6.03(3.28,8.79)	
Others	13.58(11.76,15.40)	13.58(11.59,15.56)	13.59(10.24,16.93)	
Education:				0.12
Less than high school	19.97(17.08,22.86)	19.33(16.93,21.73)	24.52(18.83,30.20)	
High school or equivalent	32.65(28.74,36.55)	32.54(29.67,35.41)	33.40(27.81,38.99)	
College or above	47.38(43.50,51.26)	48.13(45.05,51.21)	42.08(35.54,48.63)	
poverty ratio level:				**< 0.001**
0-1.0	26.36(22.46,30.26)	25.07(22.02,28.13)	35.49(31.23,39.74)	
1.1-3.0	44.51(39.85,49.16)	44.35(40.92,47.78)	45.62(40.14,51.10)	
>3.0	29.14(25.40,32.87)	30.58(26.66,34.49)	18.89(13.32,24.47)	
Marital status:				**< 0.001**
Married	52.38(47.36,57.41)	52.82(50.00,55.63)	49.32(43.83,54.81)	
Separated	22.43(19.58,25.29)	21.21(18.91,23.51)	31.12(26.33,35.91)	

Never married	25.18(22.59,27.78)	25.97(23.48,28.47)	19.56(14.69,24.44)	
Alcohol drinking:				**< 0.001**
Non-drinker	11.64(10.15,13.13)	10.45(9.09,11.81)	20.08(16.01,24.16)	
Low to moderate drinker	44.57(40.98,48.16)	44.62(42.18,47.05)	44.21(38.78,49.64)	
Heavy drinker	43.79(39.11,48.48)	44.93(42.32,47.55)	35.71(30.99,40.42)	
Leisure time physical activity level:				**< 0.001**
0 times/week	56.57(51.67,61.48)	55.02(52.51,57.53)	67.60(61.51,73.68)	
1-2 times/week	13.92(11.72,16.13)	14.80(12.60,16.99)	7.73(4.51,10.94)	
≥3 times/week	29.50(26.18,32.82)	30.18(27.57,32.80)	24.68(19.35,30.00)	
Healthy eating index score:				0.38
Quarter 1	39.69(35.54,43.85)	39.98(37.20,42.76)	37.66(32.53,42.80)	
Quarter 2	28.11(25.42,30.79)	27.87(26.28,29.47)	29.76(24.15,35.37)	
Quarter 3	21.50(19.14,23.86)	21.13(19.22,23.05)	24.09(19.53,28.64)	
Quarter 4	10.71(9.13,12.28)	11.02(9.26,12.78)	8.49(5.17,11.82)	
Self-reported health:				**< 0.001**
Very good to excellent	28.92(25.84,32.00)	26.88(24.69,29.06)	43.42(35.83,51.02)	
Good	41.44(37.32,45.57)	42.13(39.46,44.80)	36.54(30.19,42.89)	
Poor to fair	29.64(26.80,32.47)	30.99(28.51,33.47)	20.04(13.18,26.89)	
Self-reported chronic diseases:				
Diabetes	12.15(10.26,14.05)	9.69(8.20,11.18)	29.68(25.21,34.15)	**< 0.001**
Hypertension	38.80(35.01,42.60)	34.72(31.89,37.56)	67.79(62.32,73.27)	

Supplementary table 1, Baseline characteristics of CKD and non CKD populations. variables marked with '*' are represented using means and their corresponding confidence intervals, while variables marked with '#' are described using medians and quartiles. Categorical variables are presented as percentages with confidence intervals.

	Quartile	Chronic kidney disease					
		**crude model**		**Model 1**		**Model 2**	
		**OR(95%CI)**	***p***	**OR(95%CI)**	***p***	**OR(95%CI)**	***p***
Cotinine	Q1	ref		ref		ref	
	Q2	1.96(1.37,2.82)	**<0.001**	1.59(1.06,2.40)	**0.03**	1.52(0.99,2.32)	**<0.05**
	Q3	1.65(1.18,2.31)	**0.004**	1.35(0.91,2.01)	0.13	1.25(0.80,1.95)	0.31
	Q4	1.64(1.07,2.49)	**0.02**	1.26(0.79,2.00)	0.32	1.19(0.73,1.94)	0.47
	p for trend		0.09		0.67		0.91
Hydroxycotinine	Q1	ref		ref		ref	
	Q2	1.06(0.70,1.59)	0.78	0.96(0.61,1.51)	0.85	0.88(0.55,1.42)	0.59
	Q3	1.28(0.84,1.94)	0.24	1.09(0.69,1.73)	0.71	0.96(0.62,1.61)	0.85
	Q4	2.27(1.59,3.24)	**<0.001**	2.02(1.32,3.07)	**0.002**	1.61(1.06,2.43)	**0.03**
	p for trend		**<0.001**		**0.002**		**0.02**

	Quartile	Albuminuria					
		**crude model**		**Model 1**		**Model 2**	
		**OR(95%CI)**	***p***	**OR(95%CI)**	***p***	**OR(95%CI)**	***p***
Cotinine	Q1	ref		ref		ref	

	Q2	1.82(1.26,2.64)	**0.002**	1.53(1.02,2.27)	**0.04**	1.46(0.96,2.22)	0.07
	Q3	1.72(1.20,2.46)	**0.004**	1.48(1.00,2.20)	**0.05**	1.38(0.88,2.17)	0.15
	Q4	1.66(1.07,2.59)	**0.03**	1.39(0.85,2.25)	0.18	1.32(0.79,2.21)	0.28
	p for trend		**<0.05**		0.29		0.44

Hydroxycotinine	Q1	ref		ref		ref	
	Q2	1.06(0.70,1.59)	0.78	0.96(0.61,1.51)	0.85	0.88(0.55,1.42)	0.59
	Q3	1.28(0.84,1.94)	0.24	1.09(0.69,1.73)	0.71	0.94(0.62,1.64)	0.85
	Q4	2.28(1.60,3.24)	**<0.001**	2.02(1.32,3.07)	**0.002**	1.61(1.06,2.43)	**0.03**
	p for trend		**<0.001**		**0.002**		**0.02**

		**Renal insufficiency**					
	**Quartile**	**crude model**		**Model 1**		**Model 2**	
		**OR(95%CI)**	***p***	**OR(95%CI)**	***p***	**OR(95%CI)**	***p***
Cotinine	Q1	ref		ref		ref	
	Q2	1.42(1.07,1.89)	**0.02**	1.08(0.77,1.51)	0.65	1.11(0.78,1.59)	0.53
	Q3	1.84(1.36,2.50)	**<0.001**	1.42(0.96,2.11)	0.08	1.47(0.97,2.21)	0.07
	Q4	1.88(1.39,2.54)	**<0.001**	1.47(1.03,2.10)	**0.04**	1.53(1.07,2.17)	**0.02**
	p for trend		**<0.001**		**0.02**		**0.01**

	Q1	ref		ref		ref	
	Q2	1.24(0.83,1.86)	0.29	1.04(0.66,1.65)	0.85	1.10(0.69,1.77)	0.66
Hydroxycotinine	Q3	2.27(1.54,3.35)	**<0.001**	1.61(1.06,2.45)	**0.03**	1.66(1.08,2.56)	**0.02**
	Q4	2.62(1.79,3.85)	**<0.001**	2.00(1.30,3.08)	**0.003**	2.07(1.33,3.23)	**0.003**
	p for trend		**<0.001**		**<0.001**		**<0.001**

Supplementary table 2, Univariate and multivariate logistic regression analysis of cotinine(Q1, <114ng/mL; Q2, 114-211ng/mL; Q3, 212-315ng/mL; Q4, ⩾316ng/mL) and hydroxycotinine(Q1, <31ng/mL; Q2, 31-72ng/mL; Q3, 73-123ng/mL; Q4, ⩾124ng/mL) with abnormal renal function(including CKD, albuminuria, renal insufficiency), respectively. The crude model is a univariate logistic regression model; model1, adjusted for baseline age, sex, BMI, race, education level, marital status, family income- poverty ratio level, and drinking status; model2, additionally adjusted for leisure-time physical activity level, healthy eating index scores, self-reported health status and baseline history of diabetes and hypertension.

	Cotinine, ng/mL							***p for trend***	***p for interaction***
	**Q1(<114)**	**Q2(114-211)**	***p***	**Q3(212-315)**	***p***	**Q4(≥316)**	***p***		
sex									0.8
Men	ref	1.21(0.72, 2.05)	0.46	1.56(0.86, 2.82)	0.14	1.84(1.07, 3.16)	**0.03**	**0.03**	
Women	ref	1.11(0.70, 1.77)	0.65	1.59(1.00, 2.52)	**<0.05**	1.45(0.85, 2.49)	0.16	0.06	
Age, years									0.07
<60	ref	1.09(0.74,1.60)	0.64	1.82(1.18,2.81)	**0.01**	1.82(1.26,2.63)	**0.003**	**<0.001**	
≧60	ref	1.04(0.45,2.40)	0.93	0.75(0.36,1.58)	0.44	0.86(0.36,2.03)	0.72	0.54	
BMI, kg/m^2									0.57
<25.0	ref	1.16(0.57, 2.37)	0.67	1.55(0.73, 3.28)	0.24	1.39(0.63, 3.09)	0.4	0.31	
25.0-29.9	ref	1.36(0.88, 2.11)	0.15	1.17(0.65, 2.13)	0.58	1.58(0.88, 2.82)	0.12	0.18	
≧30	ref	1.02(0.63, 1.66)	0.93	1.98(1.25, 3.13)	**0.01**	1.81(1.00, 3.28)	**<0.05**	**0.002**	
Ethnicity:									0.87
Non-Hispanic white	ref	1.04(0.64, 1.69)	0.86	1.53(0.95, 2.47)	0.08	1.53(0.99, 2.35)	**<0.05**	**0.03**	
Non-Hispanic black	ref	0.96(0.51,1.80)	0.88	1.07(0.55,2.07)	0.82	1.03(0.55,1.91)	0.93	0.79	
Mexican American	ref	1.27(0.46, 3.53)	0.71	2.28(0.82, 6.33)	0.34	1.48(0.25, 8.88)	0.73	0.24	
Others	ref	1.56(0.73, 3.33)	0.24	2.06(0.90, 4.74)	0.08	3.25(1.44, 7.35)	**0.01**	**0.01**	
Education:									0.84
Less than high school	ref	0.83(0.39, 1.77)	0.61	1.17(0.46, 3.01)	0.73	1.22(0.45, 3.32)	0.69	0.45	
High school or equivalent	ref	1.54(0.79, 3.00)	0.19	2.01(1.14, 3.57)	**0.02**	1.66(0.93, 2.95)	0.08	0.08	
College or above	ref	1.02(0.59, 1.76)	0.94	1.47(0.83, 2.61)	0.17	1.75(1.08, 2.81)	**0.02**	**0.01**	
poverty ratio level:									0.11
0-1.0	ref	0.53(0.26,1.11)	0.09	1.06(0.50,2.23)	0.88	0.87(0.49,1.55)	0.63	0.56	

	Hydroxycotinine, ng/mL							*p for trend*	*p for interaction*
	**Q1(<31)**	**Q2(31-72)**	***p***	**Q3(73-123)**	***p***	**Q4(≥124)**	***p***		
1.1-3.0	ref	1.65(1.05, 2.59)	**0.03**	2.01(1.26, 3.21)	**0.01**	1.63(1.10, 2.42)	**0.02**	**0.02**	
>3.0	ref	1.11(0.57, 2.16)	0.74	1.65(0.74, 3.69)	0.21	3.04(1.39, 6.67)	**0.01**	**0.01**	
Alcohol drinking:									0.52
Non-drinker	ref	1.09(0.39, 3.04)	0.86	1.49(0.40, 5.53)	0.53	2.42(0.86, 6.78)	0.09	**0.04**	
Low to moderate drinker	ref	1.16(0.73, 1.83)	0.52	1.47(0.95, 2.26)	0.08	1.28(0.78, 2.10)	0.31	0.19	
Heavy drinker	ref	1.04(0.60, 1.79)	0.9	1.79(1.01, 3.17)	**0.05**	1.83(1.07, 3.12)	**0.03**	**0.01**	
Leisure time physical activity level:									0.87
0 times/week	ref	1.17(0.82, 1.65)	0.37	1.69(1.11, 2.58)	**0.02**	1.74(1.09, 2.78)	**0.02**	**0.02**	
1-2 times/week	ref	1.26(0.43, 3.69)	0.66	2.19(0.59, 8.05)	0.22	1.18(0.29, 4.91)	0.81	0.53	
≥3 times/week	ref	1.21(0.68, 2.17)	0.5	1.65(0.84, 3.26)	0.14	1.96(0.88, 4.35)	0.1	0.07	
Healthy eating index score:									0.35
Quarter 1	ref	0.78(0.38, 1.59)	0.48	1.77(0.96, 3.25)	0.06	1.57(0.90, 2.76)	0.11	**0.01**	
Quarter 2	ref	1.42(0.81, 2.46)	0.21	1.54(0.71, 3.31)	0.26	1.66(0.89, 3.10)	0.11	0.13	
Quarter 3	ref	0.78(0.40, 1.50)	0.44	1.07(0.49, 2.33)	0.86	1.25(0.55, 2.85)	0.58	0.46	
Quarter 4	ref	3.44(1.27, 9.36)	**0.02**	1.71(0.62, 4.71)	0.28	2.54(1.05, 6.17)	**0.04**	0.1	
Self-reported health:									0.74
Very good to excellent	ref	0.92(0.50,1.69)	0.78	1.57(0.89,2.80)	0.12	1.42(0.81,2.49)	0.21	**<0.05**	
Good	ref	1.43(0.84, 2.41)	0.17	1.70(0.87, 3.34)	0.12	1.93(1.10, 3.40)	**0.02**	**0.02**	
Poor to fair	ref	1.07(0.55, 2.08)	0.84	1.54(0.84, 2.84)	0.16	1.43(0.79, 2.60)	0.23	0.1	

sex									0.32
Men	ref	1.31(0.76, 2.27)	0.31	2.28(1.24, 4.18)	**0.01**	2.24(1.29, 3.90)	**0.01**	**0.001**	
Women	ref	1.01(0.54, 1.89)	0.98	1.43(0.82, 2.50)	0.19	2.23(1.31, 3.81)	**0.01**	**<0.001**	
Age, years									0.69
<60	ref	1.18(0.71,1.95)	0.51	1.94(1.20,3.12)	**0.01**	2.32(1.47,3.67)	**0.001**	**<0.001**	
≧60	ref	0.69(0.27,1.78)	0.43	1.12(0.57,2.20)	0.74	1.51(0.72,3.17)	0.26	**0.05**	
BMI, kg/m^2									0.76
<25.0	ref	1.06(0.43, 2.64)	0.89	1.70(0.75, 3.84)	0.19	1.65(0.72, 3.78)	0.22	0.13	
25.0-29.9	ref	1.34(0.72, 2.52)	0.34	1.94(0.95, 3.96)	0.07	2.13(1.20, 3.78)	**0.01**	**0.01**	
≧30	ref	1.08(0.65, 1.79)	0.76	1.80(1.10, 2.94)	**0.02**	3.07(1.78, 5.28)	**<0.001**	**<0.001**	
Ethnicity:									0.53
Non-Hispanic white	ref	1.00(0.52, 1.94)	1	1.53(0.86, 2.74)	0.14	1.92(1.12, 3.27)	**0.02**	**0.003**	
Non-Hispanic black	ref	0.64(0.32,1.28)	0.18	1.63(0.84,3.14)	0.13	1.61(0.85,3.04)	0.13	**0.01**	
Mexican American	ref	2.79(1.42, 5.48)	0.2	3.18(1.10, 9.20)	0.27	8.16(2.43,27.41)	0.17	**0.03**	
Others	ref	2.03(1.06, 3.91)	**0.03**	2.91(1.40, 6.05)	**0.01**	3.87(1.39,10.73)	**0.01**	**0.004**	
Education:									0.45
Less than high school	ref	1.60(0.87, 2.95)	0.12	2.23(1.10, 4.53)	**0.03**	2.95(1.48, 5.86)	**0.004**	**0.004**	
High school or equivalent	ref	1.20(0.61, 2.37)	0.57	2.17(1.22, 3.88)	**0.01**	1.72(0.99, 2.99)	**0.05**	**0.03**	
College or above	ref	0.97(0.51, 1.84)	0.92	1.46(0.79, 2.70)	0.22	2.38(1.23, 4.58)	**0.01**	**0.01**	
poverty ratio level:									0.37
0-1.0	ref	1.18(0.59,2.36)	0.63	1.47(0.83,2.61)	0.18	2.76(1.64,4.64)	**<0.001**	**<0.001**	
1.1-3.0	ref	1.33(0.76, 2.31)	0.3	1.94(1.19, 3.16)	**0.01**	2.43(1.44, 4.10)	**0.002**	**<0.001**	
>3.0	ref	0.83(0.32, 2.11)	0.68	2.00(0.87, 4.62)	0.1	1.64(0.64, 4.18)	0.29	0.1	

Alcohol drinking:									0.28
Non-drinker	ref	1.87(0.86, 4.07)	0.11	1.99(0.69, 5.73)	0.19	2.55(1.05, 6.21)	**0.04**	0.06	
Low to moderate drinker	ref	0.99(0.55, 1.78)	0.97	2.23(1.35, 3.67)	**0.003**	2.12(1.31, 3.41)	**0.004**	**<0.001**	
Heavy drinker	ref	1.06(0.59, 1.90)	0.85	1.35(0.73, 2.48)	0.32	2.16(1.20, 3.88)	**0.01**	**0.01**	
Leisure time physical activity level:									0.78
0 times/week	ref	1.04(0.66, 1.65)	0.85	1.98(1.27, 3.09)	**0.004**	2.15(1.32, 3.52)	**0.004**	**<0.001**	
1-2 times/week	ref	1.17(0.33, 4.21)	0.8	1.53(0.45, 5.16)	0.48	1.84(0.58, 5.81)	0.28	0.24	
≥3 times/week	ref	1.56(0.69, 3.50)	0.27	1.90(0.95, 3.79)	0.07	2.86(1.28, 6.41)	**0.01**	**0.01**	
Healthy eating index score:									0.95
Quarter 1	ref	0.95(0.46, 1.99)	0.89	2.00(1.18, 3.37)	**0.01**	2.03(1.09, 3.79)	**0.03**	**0.004**	
Quarter 2	ref	1.47(0.85, 2.56)	0.16	1.74(0.90, 3.34)	0.09	2.63(1.28, 5.42)	**0.01**	**0.01**	
Quarter 3	ref	0.95(0.37, 2.46)	0.91	1.26(0.60, 2.61)	0.53	2.19(0.91, 5.27)	0.08	0.07	
Quarter 4	ref	1.35(0.45, 4.04)	0.58	2.94(1.05, 8.25)	**0.04**	2.46(0.91, 6.67)	0.07	**0.04**	
Self-reported health:									0.77
Very good to excellent	ref	1.13(0.64,2.01)	0.66	1.55(0.83,2.91)	0.16	2.23(1.40,3.53)	**0.002**	**0.005**	
Good	ref	1.29(0.69, 2.39)	0.4	2.02(1.10, 3.71)	**0.03**	2.38(1.14, 4.97)	**0.02**	**0.01**	
Poor to fair	ref	0.99(0.41, 2.37)	0.98	2.04(0.96, 4.37)	0.06	1.72(0.78, 3.81)	0.17	0.06	

Supplementary table 3, subgroup analysis of serum cotinine and hydroxycotinine levels and CKD risk, adjusted for baseline age, sex, race, education level, marital status, family income- poverty ratio level, drinking and smoking status, leisure-time physical activity level, healthy eating index scores, self-reported health status, baseline history of diabetes and hypertension.

© 2025 Zhao S. et al.
